# Fluorescence polarisation activity-based protein profiling for the identification of deoxynojirimycin-type inhibitors selective for lysosomal retaining alpha- and beta-glucosidases†

**DOI:** 10.1039/d3sc01021j

**Published:** 2023-08-08

**Authors:** Daniël van der Gracht, Rhianna J. Rowland, Véronique Roig-Zamboni, Maria J. Ferraz, Max Louwerse, Paul P. Geurink, Johannes M. F. G. Aerts, Gerlind Sulzenbacher, Gideon J. Davies, Herman S. Overkleeft, Marta Artola

**Affiliations:** a Leiden Institute of Chemistry, Leiden University P. O. Box 9502 2300 RA Leiden The Netherlands h.s.overkleeft@lic.leidenuniv.nl m.e.artola@lic.leidenuniv.nl; b York Structural Biology Laboratory, Department of Chemistry, The University of York York YO10 5DD UK; c Architecture et Fonction des Macromolécules Biologiques (AFMB), CNRS, Aix-Marseille University Marseille France; d Department of Cell and Chemical Biology, Leiden University Medical Centre 2333 ZC Leiden The Netherlands

## Abstract

Lysosomal exoglycosidases are responsible for processing endocytosed glycans from the non-reducing end to produce the corresponding monosaccharides. Genetic mutations in a particular lysosomal glycosidase may result in accumulation of its particular substrate, which may cause diverse lysosomal storage disorders. The identification of effective therapeutic modalities to treat these diseases is a major yet poorly realised objective in biomedicine. One common strategy comprises the identification of effective and selective competitive inhibitors that may serve to stabilize the proper folding of the mutated enzyme, either during maturation and trafficking to, or residence in, endo-lysosomal compartments. The discovery of such inhibitors is greatly aided by effective screening assays, the development of which is the focus of the here-presented work. We developed and applied fluorescent activity-based probes reporting on either human GH30 lysosomal glucosylceramidase (GBA1, a retaining β-glucosidase) or GH31 lysosomal retaining α-glucosidase (GAA). FluoPol-ABPP screening of our in-house 358-member iminosugar library yielded compound classes selective for either of these enzymes. In particular, we identified a class of *N*-alkyldeoxynojirimycins that inhibit GAA, but not GBA1, and that may form the starting point for the development of pharmacological chaperone therapeutics for the lysosomal glycogen storage disease that results from genetic deficiency in GAA: Pompe disease.

## Abstract 2

Parallel FluoPol-ABPP screenings on lysosomal β-glucosidase (GBA1) and α-glucosidase (GAA) revealed a *N*-9-phenanthrenyl-DNJ that inhibits GAA selectively and is an interesting hit for the development of chaperones for Pompe disease.

## Graphical abstract

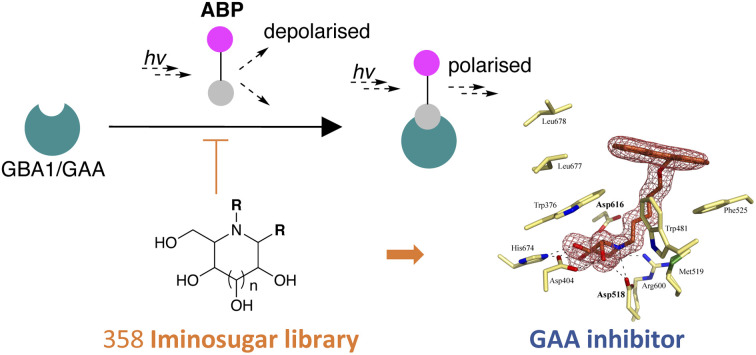

## Introduction

**Fig. 1:**
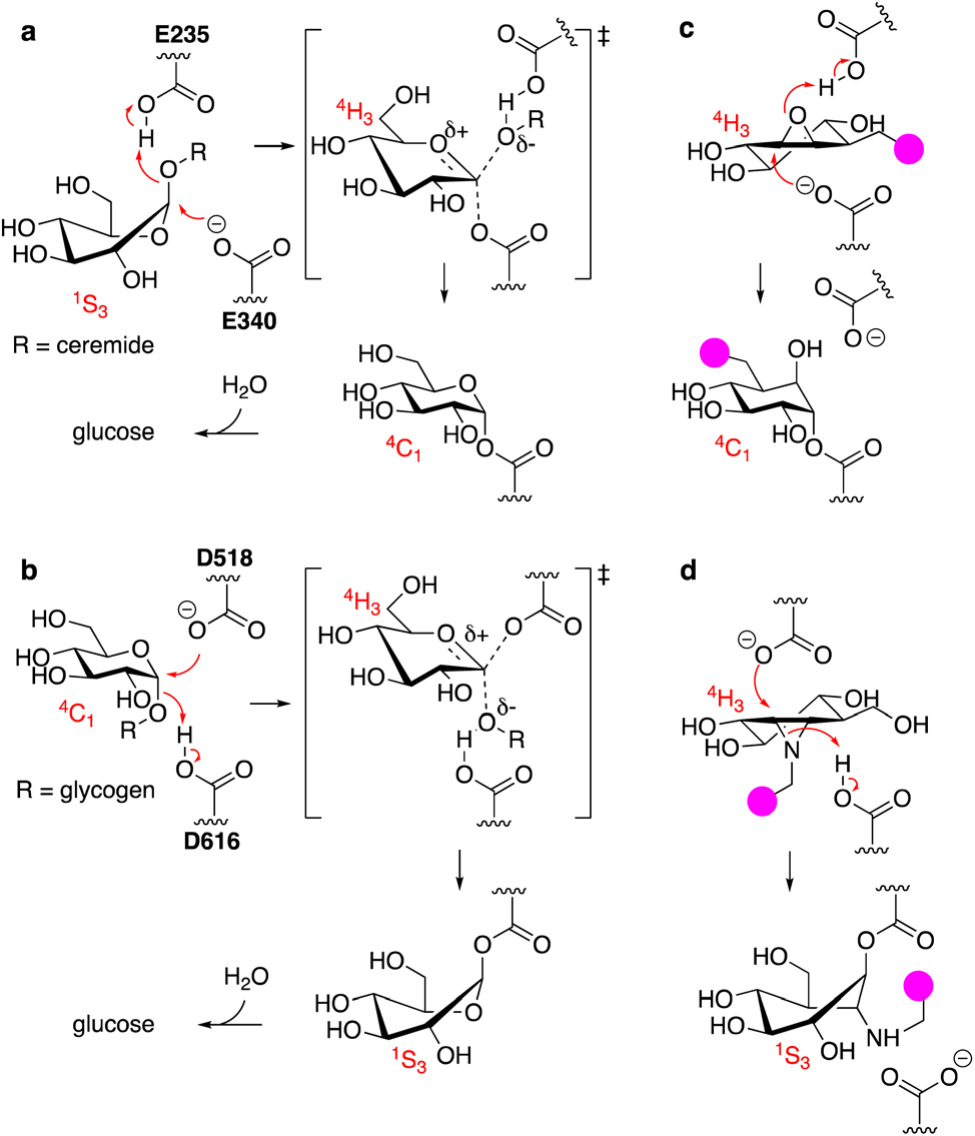
(a) Reaction itinerary employed by human GH30 lysosomal retaining β-glucosidase (GBA1) in the hydrolysis of glucosylceramide. Glu340 (E340) acts as the catalytic nucleophile and Glu235 (E235) as the catalytic acid/base. (b) Reaction itinerary employed by human GH31 lysosomal retaining α-glucosidase (GAA) in the hydrolysis of glycogen (poly-1,4/1,6-α-glucopyranose). Asp518 (D518): catalytic nucleophile; Asp616 (D616) catalytic acid/base. (c) C8-Substituted cyclophellitol covalently inhibits GH30 retaining β-glucosidases. (d) 1,6-*Epi*-cyclophellitol aziridines covalently and irreversibly inhibit GH31 α-glucosidases. Both inhibitors initially bind by virtue of their ^4^*H*_3_ transition state mimicry. Attachment of a reporter moiety (fluorophore, biotin, bioorthogonal tag – the pink bulb in c and d allows for detection and analysis of reacted proteins.

The uptake and turnover of extra- and intracellular biopolymers into their monomeric building blocks in mammals is driven by the joint action of numerous endo-lysosomal hydrolases of various families: esterases, phosphatases, phosphodiesterases, sulfatases, peptidases, proteases, and glycosidases.^1^ In all, over 70 hydrolases may be present and active at a given time in an endo-lysosomal compartment and while substrate redundancy occurs within some enzyme families, many substrates (or substrate families) are recognised and hydrolysed by one enzyme only. Deficiency in such a unique enzyme may be caused by inherited mutations in the gene encoding for the enzyme, but also by genetic mutations in genes involved in enzyme maturation or trafficking. This often results in accumulation of the underlying substrate, the more so since lysosomal enzymes often act in a conveyor-belt manner: the product of one is the substrate of the next. Substrate accumulation may cause disease and lysosomal inherited metabolic disorders are almost as numerous as lysosomal hydrolytic enzymes.^2,3^ This is well exemplified by one particular class of endo-lysosomal hydrolases: the exo-glycosidases. Polysaccharides and glycoconjugates, which constitute a major component of biopolymers that are degraded in lysosomes, may contain one or more copies of the common mammalian monosaccharides including d-glucose, d-mannose, d-galactose, d-glucosamine, d-galactosamine, l-fucose, d-glucuronic acid, l-iduronic acid, and d-neuraminic acid, often in both anomeric (alpha and beta) forms. Lysosomal exo-glycosidases are normally capable of removing one of these monosaccharides uniquely from the non-reducing end, often with little to no discrimination with regard to the aglycon but with exquisite selectivity for the configuration and substitution pattern of the monosaccharide. Deficiency in one such glycosidase results in accumulation of its substrate(s) – often oligosaccharides or oligosaccharidic glycoconjugates – simply by interrupting all downstream degradation steps. Many lysosomal storage disorders (LSDs) arise from these deficiencies, including Fabry disease,^4^ Nieman–Pick disease,^5^ Tay-Sachs disease,^6^ GM1-gangliosidosis,^7^ and the two diseases intrinsically related to the studies described here: the lysosomal glycolipid storage disorder, Gaucher disease,^8^ and the lysosomal glycogen storage disorder, Pompe disease.^9^ Gaucher disease is rooted in genetic deficiency of the retaining β-glucosidase GBA1 (glucosylceramidase, glucocerebrosidase, member of the glycoside hydrolase (GH)30 superfamily of glycoside hydrolases)^10^ and Pompe disease is caused by genetic deficiency of the retaining α-glucosidase, GAA (member of the GH31 family).^11^ GBA1 and GAA produce glucose, but do so from different substrates and by following distinct reaction itineraries.^12–14^ GBA1 hydrolyses glucosylceramide, a β-glucopyranoside, following a Koshland double displacement mechanism, the first half of which is shown in Fig. 1a. The substrate binds in a ^1^*S*_3_ conformation to position the anomeric leaving group (ceramide) in an axial orientation. Protonation of the aglycon by the active site catalytic acid-base residue and S_N_2 displacement by the active site nucleophile yields, through a flattened oxocarbenium ion-character ^4^*H*_3_ half chair intermediate, a covalent glucosyl-enzyme intermediate with the α-configured glucopyranose bound in a ^4^*C*_1_ chair conformation.

GAA processes its substrate, glycogen (α-1,4/1,6-linked glucopyranose polymers), in a related Koshland double displacement mechanism. However, and as dictated by the opposite (compared to glucosylceramide) anomeric configuration, it does so following the reverse reaction itinerary: ^4^*C*_1_ → [^4^*H*_3_]^‡^ → ^1^*S*_3_ (Fig. 1b). Both GBA1 and GAA-mediated hydrolyses proceed through a flattened ^4^*H*_3_ transition state, a feature that, in combination with the occurrence of a covalent intermediate, can be capitalised on for the design of covalent inhibitors and activity-based probes (ABPs).^15–17^ Cyclophellitol, a natural product cyclitol epoxide with a configuration emulating a β-glucopyranoside, is a potent mechanism-based retaining β-glucosidase inhibitor.^18,19^ Grafting a reporter moiety at C8 (cyclophellitol numbering) delivers a selective and effective GBA1 ABP that reacts within the active site as shown in Fig. 1c.^16^ In contrast to most exo-glycosidases, GBA1 does accept substrates^20,21^ and inhibitors featuring functional groups appended to the glucopyranose (-like) ring at positions other than the anomeric one, explaining the exquisite selectivity of C8-substituted cyclophellitols.^17^ 1,6-*Epi*-cyclophellitol in turn is a mechanism-based retaining α-glucosidase inhibitor.^22^ Substitution of the primary alcohol in 1,6-*epi*-cyclophellitol for a reporter is not an option – inhibitory potency will be lost – and the same holds true for any of the three secondary alcohols. Substitution of the epoxide oxygen for an aziridine nitrogen and installation of a reporter on this nitrogen, as depicted in Fig. 1d, does yield an effective activity-based probe that, while not GAA selective, is in general in-class selective for retaining α-glucosidases.^23^

We have recently reported on the development of FluoPol-ABPP assays for the identification, from our iminosugar library, of inhibitors selective for the non-lysosomal glucosylceramidase (GBA2)^24^ and Golgi mannosidase II (GMII).^25^ Although such high-throughput screenings can, in principle, also be performed with acid-sensitive fluorogenic substrates when using purified protein, we have demonstrated in the past that FluoPol-ABPs can be employed in complex cell lysates provided that a selective ABP is available.^24^

**Fig. 2:**
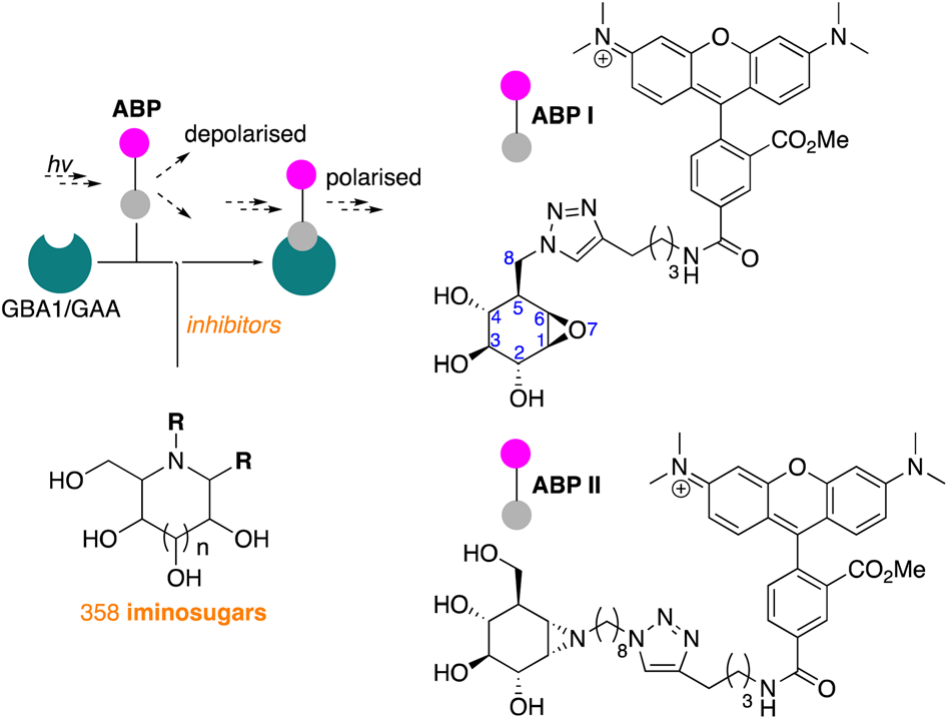
Fluorescence polarisation activity-based protein profiling (FluoPol-ABPP) screening of our 358-compound iminosugar library on rhGBA1 (Cerezyme®) and rhGAA (Myozyme®) subject of the here presented studies, and structures of the TAMRA-ABPs I and II used in the FluoPol-ABPP screenings.

In line with literature studies on FluoPol-ABPP on other hydrolases, we employed tetramethylrhodamine (TAMRA) probes for fluorescent readout and created ABPs I and II (Fig. 2), the synthesis of which is described here. Further outlined in this paper are the identification, from our libraries, of GBA1 and GAA inhibitors and comparison of the two datasets yields a set of hydrophobic, *N*-alkyl-deoxynojirimycin derivatives that inhibit GAA, but not GBA1. The binding-mode of one such compound, which we consider a potential lead for the development of pharmacological chaperones for Pompe disease, is provided by means of a co-crystal structure where the inhibitor is bound to the active site of human GAA.

## Results and discussion

### GBA1 screen

**Fig. 3:**
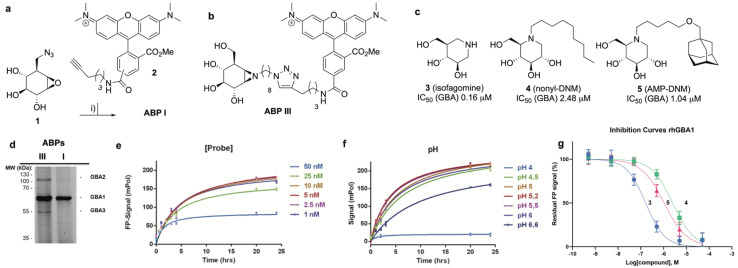
(a) Synthesis of GBA1 ABP I. Reagents and conditions: (i) mixture of 5′-and 6′-TAMRA-alkyne (2), sodium ascorbate, CuSO_4_, ^*t*^BuOH/toluene/H_2_O (1 : 1 : 1 v : v : v), 18 h at ambient temperature, 20% yield. (b) Chemical structure of ABP III. (c) Competitive inhibitors used for validation of the FluoPol ABPP assay on GBA1. Apparent inhibitory potency (IC_50_) values given are derived from competitive ABPP on rhGBA1 using ABP I as the readout. (d) In-gel fluorescence reveals GBA1 selectivity of ABP I compared to broad-spectrum retaining β-glucosidase ABP III in a 1 : 1 mixture of HEK293T GBA2 overexpressing and HEK293T GBA3 overexpressing cell lysates (labeling with 500 nM probe). (e) and (f) Optimization of ABP I concentration (e) and pH (f). (g) Inhibition curves of 3 (blue line), 4 (green line) and 5 (pink line) as determined by FluoPol-ABPP.

The synthesis of TAMRA-ABP I (Fig. 3a) was accomplished by copper(i)-catalysed [2 + 3] azide–alkyne cycloaddition of 8-azido-cyclophellitol (1), the synthesis of which we reported previously,^26^ with TAMRA-alkyne (2). ABP I modifies exclusively GBA1 in mouse brain extracts without binding the other two retaining β-glucosidases expressed in this tissue, GBA2 and GBA3, which are targeted by broad-spectrum retaining β-glucosidase ABP III (ref. 24) (previously developed to perform FluoPol-ABPP on recombinant GBA2, Fig. 3b and d). In this respect, and as expected from crystallographic studies (See Fig. S1–S4† for Bodipy-tagged GBA1 ABP IV), TAMRA-ABP I behaves as our previously reported Bodipy-tagged selective GBA1 probes^16^ and is thus in principle suited for FluoPol-ABPP-based discovery of GBA1 inhibitors (Fig. 3d and S6†).

Development of the FluoPol-ABPP assay was conducted in 384-well format (*V*_final_ = 25 μL) using 2 μg mL^−1^ (36 nM) recombinant human GBA1 (rhGBA1, Cerezyme®). All the biochemical reactions were performed in 150 mM McIlvaine buffer supplemented with 0.2% (w/v) sodium taurocholate and 0.1% (v/v) Triton X-100. In order to discriminate between enzyme-bound and unbound probe (detected as high and low FluoPol-signal), the reaction conditions were optimized. The highest polarization signal was obtained when incubating 5–25 nM probe (Fig. 3e) at pH = 5.2 (Fig. 3f) for 24 h. The observed optimal pH is consistent with the pH optimum for GBA1. The FluoPol-signal started to decrease upon surpassing the 1 : 1 enzyme-ABP ratio as the excess of free ABP can decrease the fluorescence polarization (Fig. 3e and S7†). Subsequently, three GBA1 inhibitors with varying potency, namely isofagomine (3), *N*-nonyl-deoxynojirimycin (4) and *N*-(5-adamantane-1-yl-methoxy-pentyl)-deoxynojirimycin (AMP-DNM, 5) were tested in the assay. The inhibitors were pre-incubated at 37 °C with rhGBA1 at 36 nM for 1 hour before addition of ABP I at the same concentration.

The residual FP-signal was measured after 24 h probe incubation at 37 °C. The competitors showed a dose-dependent response and the apparent half maximal inhibitory concentration (IC_50_) values of 3, 4 and 5 are 0.16, 2.48 and 1.04 μM, respectively (Fig. 3g). Although the trend in potency is in accordance to the literature IC_50_ values, our the inhibitory potencies are higher compared the reported values,^27^ which were determined using a conventional competitive fluorogenic substrate assay (hydrolysis of 4-methylumbelliferyl-β-glucopyranoside). This can be explained by the covalent nature of ABP I, which can strongly compete and displace competitive iminosugars. Therefore, the concentration of ABP I influences the resulted apparent IC_50_ value. Nevertheless, our FluoPol-ABPP assay appears suited for the screening of GBA1 inhibitors from larger compound collections.

**Fig. 4:**
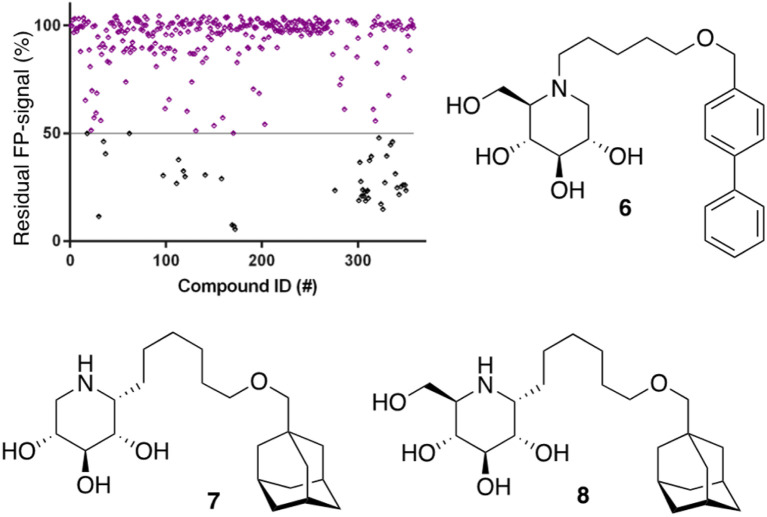
Screening of our in-house iminosugar library containing 358 entries at a concentration of 5 μM in the FluoPol-ABPP assay on rhGBA1. 38 Compounds showed more than 50% reduction in ABP I FP-signal. Representative inhibitors include compounds 6, 7 and 8.

After optimization of the reaction conditions the assay volume was miniaturized into 15 μL, and our iminosugar compound library screened at a final inhibitor concentration of 5 μM. Based on the apparent IC_50_ values obtained with ABP I and to ensure the identification of significant hits, we decided to lower the concentration of ABP to 5 nM in our HTS. Of the 358 compounds tested in the competitive FluoPol-ABPP assay, 38 showed more than 50% reduction in FP-signal at 5 μM (Fig. 4). These hit compounds can be divided in two groups; iminosugars containing a biphenyl moiety (exemplified by compound 6) and two types of α-aza-*C*-glycosides: one featuring the *xylo*-deoxynojirimycin configuration, as in 7, and one featuring deoxynojirimycin configuration, as in 8 (Fig. 4). The iminosugars with differently substitute biphenyl moieties are known nanomolar glucosylceramide synthase (GCS) and GBA2 inhibitors.^27^ More than 30 of such dual GCS/GBA2 inhibitors have been reported to also inhibit GBA1 at micromolar levels. The GBA1 inhibitory potency of α-aza-C-glycosides 7 and 8 as determined from the competitive FluoPol-ABPP assay (>90% inhibition) is consistent with the IC_50_ values we reported previously.

### GAA screen

**Fig. 5:**
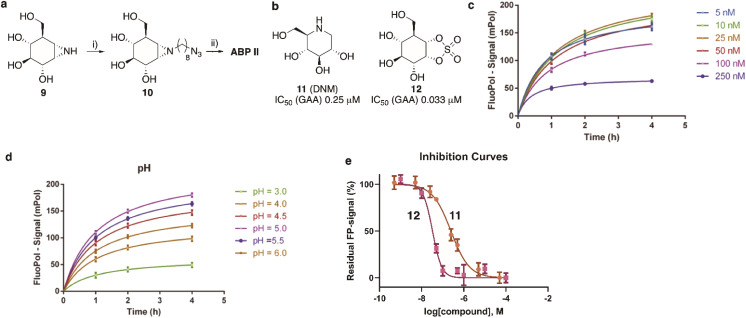
(a) Synthesis of GAA ABP II. Reagents and conditions: (i) 1-azido-8-iodooctane, K_2_CO_3_, DMF, 80 °C, 39% yield; (ii) mixture of 5′/6′-tetramethylrhodamine-1-pentyne-amide, sodium ascorbate, CuSO_4_, DMF, 3 days at ambient temperature, 21% yield. (b) Competitive (DNM, 11) and covalent irreversible (1,6-*epi*-cyclophellitol cyclosulfate, 12) inhibitors used for validation of the FluoPol-ABPP assay on GAA. Apparent IC_50_ values are derived from competitive ABPP on rhGAA using ABP II as the readout. (c) and (d) Optimization of ABP II concentration (c) and pH (d). (e) Fluorescent polarisation competition curves of 11 (brown line) and 12 (magenta line) as determined by FluoPol-ABPP.

The synthesis of TAMRA-ABP II (Fig. 2) commenced with 1,6-*epi*-cyclophellitol aziridine 9, which we prepared following our previously reported procedures (Fig. 5a).^23^ The aziridine in 9 was subsequently reacted with 1-iodo-8-azido-octane in DMF and with potassium carbonate as the base, yielding compound 10. Ensuing copper(i)-catalysed azide–alkyne [2 + 3] cycloaddition with TAMRA-alkyne yielded GAA ABP II.

As the next objective, optimal FluoPol-ABPP conditions for screening our iminosugar compound library were sought for, with respect to probe-to-enzyme ratio, to pH optimum and whether such optimal conditions would return viable inhibitory data – data that match those obtained in orthogonal assays using known GAA inhibitors. The reaction conditions for probe labelling on recombinant human GAA (rhGAA, Myozyme®) were first optimized in a 384-well format (*V*_final_ 25 μL). Biochemical assays were conducted with 8.45 μg mL^−1^ (80 nM) rhGAA in 150 mM McIlvaine buffer (supplemented with 0.1% bovine gamma globulin and 0.5 mg mL^−1^ CHAPS). An optimal FluoPol signal was obtained when incubating rhGAA (80 nM) with 5 to 50 nM probe II (Fig. 5c) at pH 5.0 (Fig. 5d – matching the pH optimum of this lysosomal enzyme) for 4 hours. Pre-incubation of rhGAA at 80 nM with deoxynojirimycin (11, DNM), a known reversible GAA inhibitor, or 1,6-*epi*-cyclophellitol cyclosulfate (12),^12^ a known irreversible GAA inhibitor, led to dose-dependent competition with ABP II (also at 80 nM) (Fig. 5b and e). In this assay DNJ 11 inhibits rhGAA in the high nanomolar range, whereas cyclosulfate 12 fully inactivates the enzyme at 33 nM. These observed apparent IC_50_ values are consistent with our previous findings obtained from a gel-based competitive ABPP assay.^12,28^

**Fig. 6:**
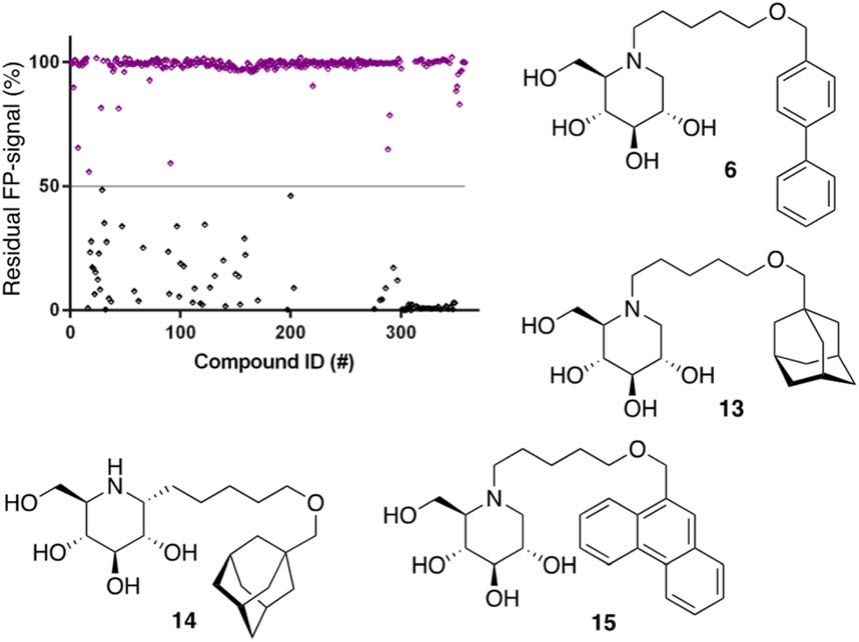
Screening of our in-house iminosugar library containing 358 entries at a concentration of 5 μM in the FluoPol-ABPP assay on rhGAA. Approximately 80 compounds showed more than 50% reduction in ABP II FP-signal. Representative inhibitors include compounds 6 and 13–15.

Following the above-described assay optimization, our in-house 358-entry iminosugar library was screened in FluoPol-ABPP format at a concentration of 5 μM (*V*_final_ = 15 μL) using 25 nM of ABP II. The compounds were plotted against the residual FP-signal, as shown in Fig. 6. Approximately 80 compounds show more than 50% reduction in ABP II FP-signal (see for all structures and activities the ESI†). With the aim to look further into this, we assessed the 80 hits from the FluoPol-ABPP screen in an orthogonal, fluorogenic substrate (methylumbelliferyl-α-glucoside) assay against rhGAA and also ER-α-glucosidase II (GANAB), an enzyme involved in protein quality control in the endoplasmic reticulum. The results of these measurements are given in Table S2.† Several relevant structures (compounds 6 and 13–15) are depicted in Fig. 6. All selected compounds inhibited GANAB with similar potencies as for rhGAA with the exception of compound 14. Compounds 6 and 13 are nanomolar inhibitors of the glucosylceramide metabolizing enzymes, GCS and GBA2 and also inhibit the Gaucher enzyme, GBA1, in the micromolar range (Table 1). Aza-C-glycoside 14 inhibits GBA1 in the nanomolar range as well, whereas *N*-alkyl 15 bearing a branched 9-phenanthrenyl moiety displays modest activity against GCS and GBA1. This compound would therefore represent an attractive scaffold for the development of dual GAA and GBA2 inhibitors.

**Table 1:** IC_50_ values (μM) for *in vitro* inhibition of human recombinant lysosomal α-glucosidase GAA (Myozyme), ER α-glucosidase GANAB (from Pompe disease Fibroblast lysates), β-glucosidases rhGBA1 (Cerezyme) and GBA2 (GBA2-overexpressing HEK293T lysate), and *in situ* cell inhibition of glucosylceramide synthase (GCS) (RAW 264.7 cells). Reported values are mean ± standard deviation from 3 technical replicates (Fig. S8)

Cmp	GAA	GANAB	GBA1	GBA2	GCS
6	1.18 ± 0.31	8.29 ± 0.17	0.40^a^	0.002^a^	0.05^a^
13	1.39 ± 0.26	5.36 ± 0.57	0.10^a^	0.002^a^	0.2^a^
14	4.00 ± 0.83	>100	0.07^a^	0.300^a^	50^a^
15	0.11 ± 0.05	1.96 ± 0.04	10^b^	0.10^b^	>20^b^

a Values from ref. 24.

b Values from ref. 27.

**Fig. 7:**
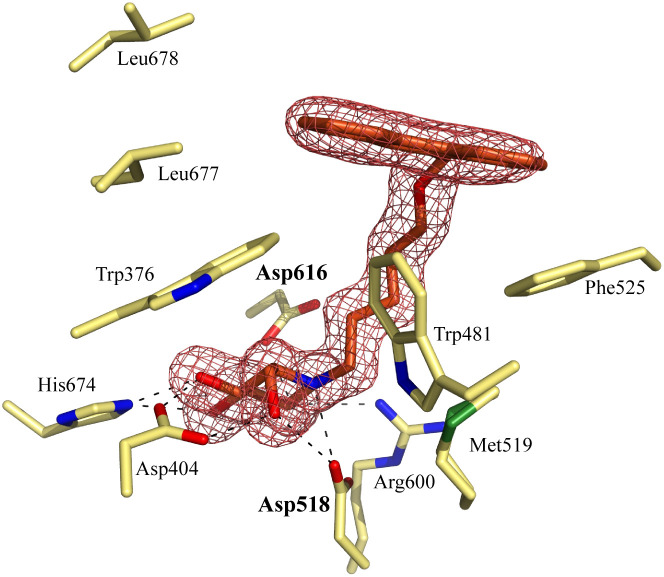
Crystal structure of rhGAA in complex with branched *N*-PNT-DNM 15. The iminosugar moiety of 15 binds in a ^4^*C*_1_ conformation. Electron density map (2*F*_o_ − *F*_c_) contoured at 1.0*σ* (0.06 e Å^−3^).

Notably, some compounds we identified as rhGAA inhibitors have not emerged in the GBA1 screen described above. Of the thus identified rhGAA *versus* GBA1-selective inhibitors, we found compound 15 (Fig. 6) of particular interest. It features a branched apolar phenanthrenyl (PNT) group, which apparently negatively impacts GBA1/GCS inhibition and it might be worthwhile to explore this design in more depth with the aim to identify selective GAA inhibitors. With the aim to prepare for such future studies, and with the thought that structural features of branched *N*-PNT-DNM 15 may be further optimized in a structure-assisted rational design program, we sought a co-crystal structure of a proteolytically digested form of rhGAA (Myozyme®) with compound 15. In the crystal structure of the complex, obtained at 1.75 Å resolution (Fig. 7), the iminosugar component is accommodated in the rhGAA active site, and overlaps virtually with DNJ as observed in the previously reported crystal structure of rhGAA in complex with this reversible inhibitor (PDB 5NN5).^29^ By this virtue, the iminosugar moiety adopts a ^4^*C*_1_ conformation and its hydroxyl groups interact *via* hydrogen bonds with rhGAA residues Asp404, Asp616 (the acid/base), His674, Arg600, and by solvent mediated interactions with Asp443, Asp645, Trp613, and Trp481. The rhGAA nucleophile Asp518 interacts tightly with the iminosugar endocyclic nitrogen. Overall, the sum of all these energetically favourable interactions testifies the inhibitory efficacy of iminosugar based inhibitors towards GAA. Beyond the iminosugar moiety, the alkyl chain of *N*-PNT-DNM 15 establishes hydrophobic interactions with the rhGAA side-chains of Met519, Trp376, Trp481, and Phe525. Finally, the side-chains of Phe525, Trp376 and the rim of the indole ring of Trp481 provide a hydrophobic stacking platform for the phenanthrenyl moiety of compound 15, which in turn makes further hydrophobic contacts with Leu677 and Leu678, located at the entry of the large groove leading to the rhGAA active site. Noteworthy, the presence of the *N*-alkyl chain and the phenanthrenyl moiety of compound 15 induce structural rearrangements of the side chains of Trp367, Trp481 and Phe525 with respect to the structure of unliganded rhGAA (PDB 5NN3),^29^ the three residues thereby approaching the ligand and strengthening the hydrophobic interactions. Most of the amino acids interacting with the ligand are conserved in human GANAB, with the exception of rhGAA Leu678, replaced by an Asp in GANAB, which might explain the similar, but slightly lower inhibition potency of compound 15 towards GANAB. The other enzymes probed for *N*-PNT-DNM 15 within this study, GBA1 (GH30), GBA2 (GH116) and GCS (GT21) are structurally too distant from GAA to draw structure-guided conclusions on inhibitor selectivity.

## Conclusions

In conclusion, the work described in this paper underscores our previous findings^24,25^ on the use of FluoPol-ABPP as a means to discover lead-inhibitors for retaining exoglycosidases. The combination of cyclophellitol-based probes and focused glycomimetic libraries provide powerful means to discover new leads for drug discovery programs, as here demonstrated for the identification of the selective (with respect to GBA1) competitive GAA inhibitor 15. Recent work from our groups on a different class of competitive GAA inhibitor (1,6-*epi*-cyclophellitol cyclosulfamidate)^28^ complements earlier studies by others^30–32^ in demonstrating, in animal models, the potential of competitive inhibitors for pharmacological chaperone therapy (PCT) for the treatment of Pompe disease. The current thinking is that PCT in combination with enzyme replacement therapy (ERT – intravenous administration of rhGAA), in which the pharmacological chaperone stabilises recombinant enzyme in circulation, is realistic. Furthermore, PCT as a standalone treatment modality to stabilize, in cells, genetically impaired GAA such that active copies reach their destination (lysosomes) may also be feasible, and for both therapeutic strategies suitably potent and selective, competitive GAA inhibitors are required. Our finding that some branched *N*-alkyl-deoxynojirimycins are selective GAA inhibitors *versus* GBA1 and GCS, combined with the structural data on the binding mode of one of these to rhGAA may pave the way for structure-guided rational design towards such compounds. More broadly speaking, the work described here also reveals that substitution of the Bodipy-type dyes we routinely install on our cyclophellitol ABPs as reporter entities, for the TAMRA dye (commonly used in FluoPol-based assays) is not detrimental for probe affinity. We confirmed this also by crystal structures, with as examples (in the ESI†) the covalent complex of rhGBA1 reacted with a previously reported Bodipy variant of ABP I as well as that of rhGAA reacted with TAMRA-ABP II. FluoPol-ABPP, and ABPP in general, thus remains an attractive technology in chemical biology and medicinal chemistry, but the technique relies on the availability of suitable mechanism-based enzyme inhibitors. Cyclophellitol derivatives provide such inhibitors for retaining exoglycosidases, and also endoglycosidases, however related compounds for other glycoprocessing enzymes including inverting glycosidases and glycosyltransferases do not exist yet. Our current research efforts, besides exploiting the results presented here, are therefore focused in this direction: the discovery of covalent inhibitors and ABPs for glycoprocessing enzymes for which no such compounds have been described to date.

## Experimental

### Synthesis

All reagents were of a commercial grade and were used as received unless stated otherwise. All reactions were performed under an argon atmosphere unless stated otherwise. Solvents used for flash column chromatography were of pro analysis quality. Reactions were monitored by analytical thin-layer chromatography (TLC) using Merck aluminum sheets pre-coated with silica gel 60 with detection by UV absorption (254 nm) and by spraying with a solution of an aqueous solution of KMnO_4_ (7%) and K_2_CO_3_ (2%) followed by charring at *ca.* 150 °C. ^1^H NMR and ^13^C NMR spectra were recorded on Bruker AV-III-HD-850 (850/214 MHz) spectrometer in the given solvent. Chemical shifts are given in ppm relative to the chloroform residual solvent peak or tetramethylsilane (TMS) as internal standard. Coupling constants are given in Hz. All given ^13^C spectra are proton decoupled. 2D NMR experiments (HSQC, COSY and NOESY) were carried out to assign protons. High-resolution mass spectra (HRMS) were recorded with an apex-QE instrument (Bruker). For reversed-phase HPLC-MS purifications an Agilent Technologies 1200 series prep-LCMS with a 6130 Quadropole MS system was used equipped with buffers A: 50 mM NH_4_HCO_3_ in H_2_O and B: MeCN.

ABP III,^24^ABP IV,^16^ 8-deoxy-8-azido-cyclophellitol 1 (ref. 16 and 26) and (1*S*,2*S*,3*S*,4*R*,5*R*,6*S*)-7-(8-azidooctyl)-5-(hydroxymethyl)-7-azabicyclo[4.1.0]heptane-2,3,4-triol 10 (ref. 23) were synthesized following procedures previously described and their spectroscopic data are in agreement with those previously reported. All final compounds were lyophilized and aliquoted in 100–1000 nmol tubes before tested. 4MU-α-glucopyranoside and 4MU-β-glucopyranoside were obtained from Sigma-Aldrich.

#### TAMRA-*epi*-cyclophellitol aziridine ABP II

Azido intermediate 1 (ref. 16 and 26) 6 mg, 0.03 mmol, 1 equiv. and 5’/6′-tetramethylrhodamine-1-pentyne-amide (15.2 mg, 0.03 mmol, 1 equiv.) were dissolved in ^*t*^BuOH/toluene/H_2_O (3 mL, 1 : 1 : 1, v/v/v). CuSO_4_ (0.06 mL, 0.1 M in H_2_O) and sodium ascorbate (0.06 mL, 0.1 M in H_2_O) were added, and the reaction mixture was stirred at room temperature for 18 h under argon atmosphere. Then, the solution was diluted with CH_2_Cl_2_, washed with H_2_O, dried over MgSO_4_ and concentrated under reduced pressure. The crude was purified by silica gel column chromatography (CH_2_Cl_2_ to CH_2_Cl_2_/MeOH 9 : 1), subsequently purified by semipreparative reversed-phase HPLC (linear gradient: 24% to 28% B in A, 12 min, solutions used A: 50 mM NH_4_HCO_3_ in H_2_O, B: MeCN) and lyophilized to yield ABP I as 5′-TAMRA (4.3 mg, 6.0 μmol, 20%) and a smaller fraction of and 6′-TAMRA cyclophellitol (1.9 mg, 2.7 μmol, 9%) was also isolated. Both isomers presented similar labelling properties.

#### 5′-TAMRA ABP I

^1^H NMR (600 MHz, MeOD): *δ* 8.50 (d, *J* = 1.9 Hz, 1H), 8.03 (dd, *J* = 7.9, 1.8 Hz, 1H), 7.84 (s, 1H), 7.35 (d, *J* = 7.9 Hz, 1H), 7.25 (d, *J* = 9.4 Hz, 2H), 7.02 (dd, *J* = 9.4, 2.5 Hz, 2H), 6.92 (d, *J* = 2.4 Hz, 2H), 4.80 (dd, *J* = 13.9, 3.7 Hz, 1H, rotamer), 4.61 (dd, *J* = 14.0, 8.5 Hz, 1H rotamer), 3.61 (d, *J* = 8.2 Hz, 1H), 3.48 (t, *J* = 6.9 Hz, 2H), 3.28 (s, 12H), 3.23 (dd, *J* = 10.0, 8.1 Hz, 1H), 3.13 (t, *J* = 9.8 Hz, 1H), 3.05 (d, *J* = 3.9 Hz, 1H), 3.02 (t, *J* = 2.9 Hz, 1H), 2.81 (t, *J* = 7.4 Hz, 2H), 2.43–2.36 (m, 1H), 1.85–1.79 (m, 2H), 1.77–1.67 (m, 2H). ^13^C NMR (150 MHz, MeOD): *δ* 169.1, 162.1, 159.1, 158.7, 148.9, 137.3, 136.9, 132.6, 130.8, 129.5, 129.4, 124.4, 115.0, 114.8, 97.3, 78.3, 72.6, 68.7, 57.6, 55.6, 50.7, 49.6, 49.5, 49.4, 49.3, 49.2, 49.0, 48.9, 48.7, 48.6, 44.7, 40.8, 40.7, 29.8, 27.8, 25.9. HRMS: found 711.3140 [M + H]^+^, calculated for [C_38_H_43_O_8_N_6_ + H]^+^ 711.3142.

#### 6′-TAMRA ABP I

^1^H NMR (600 MHz, MeOD): *δ* 8.14 (d, *J* = 8.1 Hz, 1H), 8.07 (dd, *J* = 8.2, 1.8 Hz, 1H), 7.78 (s, 1H), 7.69 (s, 1H), 7.27 (d, *J* = 9.4 Hz, 2H), 7.03 (dd, *J* = 9.5, 2.5 Hz, 2H), 6.94 (d, *J* = 2.4 Hz, 2H), 4.77 (dd, *J* = 13.7, 3.9 Hz, 1H rotamer), 4.56 (dd, *J* = 13.9, 8.7 Hz, 1H rotamer), 3.59 (d, *J* = 8.2 Hz, 1H), 3.41 (t, *J* = 6.9 Hz, 2H), 3.29 (s, 12H), 3.21 (dd, *J* = 10.0, 8.1 Hz, 1H), 3.11 (t, *J* = 9.8 Hz, 1H), 2.75 (t, *J* = 7.5 Hz, 2H), 2.38–2.33 (m, 1H), 1.77–1.71 (m, 2H), 1.70–1.63 (m, 2H), 1.30–1.28 (m, 2H); ^13^C NMR (151 MHz, CD_3_OD): *δ* 168.3, 161.9, 158.7, 158.4, 148.4, 144.0, 136.1, 133.6, 132.4, 130.6, 129.1, 123.9, 114.7, 114.6, 96.9, 77.9, 72.1, 68.3, 57.2, 55.1, 50.3, 49.2, 49.0, 48.9, 48.7, 48.6, 48.5, 48.3, 48.2, 48.1, 44.3, 40.4, 40.4, 29.3, 27.4, 25.4; HRMS: calcd for C_38_H_43_N_6_O_8_ [M + H]^+^ 711.31424, found: 711.31400.

#### TAMRA-*epi*-cyclophellitol aziridine ABP II

Azido intermediate 10 (ref. 23) (5 mg, 15 μmol) was dissolved in DMF (2 mL). Subsequently CuSO_4_ (1 M, 34 μL, 34 μmol) and sodium ascorbate (1 M, 34 μL, 34 μmol) were added to the solution under argon atmosphere. Then, a solution of 5′/6′-tetramethylrhodamine-1-pentyne-amide (5.9 mg, 11 μmol) in 1 mL of DMF was added and the reaction mixture was stirred at room temperature for 3 days. Then, the mixture was concentrated under reduced pressure and purified by semi-preparative reversed HPLC (linear gradient: 25% to 37% B in A, 12 min, solutions used A: 50 mM NH_4_HCO_3_ in H_2_O, B: MeCN), the fractions were concentrated and lyophilized to a deep purple powder product as a mixture of 5′- and 6′-TAMRA ABP II (1.96 mg, 2.31 μmol, 21%). ^1^H NMR (850 MHz, MeOD) *δ* 8.77 (d, *J* = 1.8 Hz, 1H), 8.56 (s, 1H), 8.31 (dd, *J* = 7.9, 1.9 Hz, 1H), 7.95 (dd, *J* = 8.3, 1.4 Hz, 1H), 7.81 (s, 1H), 7.58 (d, *J* = 7.9 Hz, 1H), 7.44–7.39 (m, 1H), 7.38–7.33 (m, 1H), 7.14 (d, *J* = 9.5 Hz, 2H), 7.09 (dd, *J* = 9.5, 2.5 Hz, 2H), 7.04 (d, *J* = 2.5 Hz, 2H), 6.68 (d, *J* = 8.8 Hz, 1H), 6.51 (d, *J* = 2.6 Hz, 1H), 6.48 (dd, *J* = 8.9, 2.6 Hz, 1H), 4.66 (br s, 2H), 4.39 (t, *J* = 7.1 Hz, 2H), 3.87 (dd, *J* = 10.7, 3.9 Hz, 1H), 3.67–3.65 (m, 1H), 3.62 (dd, *J* = 10.8, 7.2 Hz, 1H), 3.53 (t, *J* = 7.0 Hz, 1H), 3.35–3.31 (m, 13H), 3.27–3.23 (m, 1H), 3.06 (t, *J* = 10.0 Hz, 1H), 2.97 (s, 1H), 2.81 (t, *J* = 7.5 Hz, 2H), 2.37–2.30 (m, 1H), 2.18 (s, 1H), 2.17–2.13 (m, 1H), 1.91 (s, 3H), 1.87–1.80 (m, 2H), 1.78–1.73 (m, 2H), 1.70–1.66 (m, 2H), 1.62–1.56 (m, 2H), 1.39–1.30 (m, 6H). ^13^C NMR (214 MHz, MeOD) *δ* 178.9, 166.5, 164.9, 160.1, 158.5, 157.7, 147.4, 136.5, 131.2, 130.7, 130.5, 130.4, 129.9, 129.6, 128.8, 127.3, 124.9, 121.9, 114.3, 113.2, 96.1, 74.3, 71.9, 71.0, 62.1, 60.8, 51.7, 49.9, 48.0, 45.5, 44.6, 40.5, 39.6, 39.5, 29.9, 29.1, 29.0, 28.5, 28.5, 26.9, 26.6, 26.0, 24.5, 23.4, 22.8. HRMS found 852.4639 [M]^+^, calculated for [C_47_H_63_O_8_N_7_]^+^ 852.4654.

### SDS-gel activity-based protein profiling experiments on tissues

Lysates of HEK293T GBA2 overexpressing and HEK293T GBA3 overexpressing cells^24^ were prepared in 150 mM McIlvain buffer (pH = 5.0), supplemented with 0.25 M sucrose, 0.2% sodium taurocholate (w/v) and 0.1% Triton X-100 (v/v). The lysates were homogenized using sonication, after which the total protein concentration was determined *via* Pierce BCA Protein assay (ThermoFisher) using BSA (ThermoFisher) for standards. Samples (*V*_f_ = 20 μL) of an 1 : 1 mixture of GBA2 : GBA3 lysates containing 40 μg protein were incubated for 30 min at 37 °C with 500 nM ABP. Protein content was denatured using Laemmli Buffer (4×) at 98 °C for 5 min. Reactions were resolved by 7.5% SDS-PAGE electrophoresis and wet slabs were scanned for fluorescence (Typhoon FLA 9500, GE Healthcare).

### Optimization of the FluoPol-ABPP assay

Recombinant human rhGBA1 (Cerezyme® from Genzyme) was used during FluoPol-ABPP assays. The optimal probe concentration on FluoPol signal was determined by varying ABP I concentrations from 1 nM to 50 nM probe at a constant protein concentration (2 μg mL^−1^, 36 nM) at pH 5.2. FluoPol-ABPP assays were also performed at different pH values at optimal probe concentration (5 nM) and rhGBA1 at 36 nM. Competition experiments were conducted by 1 hour pre-incubation of compounds in the protein solution at 37 °C (2.5% DMSO). All reactions (*V*_final_ = 25 μL) were supplemented with 0.2% sodium taurocholate (w/v), 0.1%, Triton X-100 (v/v) and were carried out in 384-wells plates (small-volume black, Greiner). FluoPol-signals were monitored on a CLARIOstar Plus (BMG Labtech) using *λ*_ex_ 540 nm and *λ*_em_ 590 nm. Samples containing an excess of isofagomine were used as reference samples (0% probe labelling), samples without inhibitors for 100% labelling controls and samples without probe as blanks to correct for background polarization.

Recombinant human rhGAA (Myozyme® from Genzyme) was used during FluoPol-ABPP assays. The optimal probe concentration on FluoPol signal was determined by varying ABP II concentrations from 1 nM to 250 nM probe at a constant protein concentration (8.45 μg mL^−1^, 80 nM) and at pH = 5.0. FluoPol-ABPP assays were also performed at different pH values by preparation of different McIlvaine buffers, supplemented with 0.5 mg mL^−1^ Chaps. These pH-experiments were performed at optimal probe concentration (25 nM) and 80 nM of rhGAA. Competition experiments were conducted by 1 hour pre-incubation of compounds in the protein solution at 37 °C (2.5% DMSO). All reactions (*V*_final_ = 25 μL) were carried out in 384-wells plates (small-volume black, Greiner). FluoPol-signals were monitored on a CLARIOstar Plus (BMG Labtech) using *λ*_ex_ 540 nm and *λ*_em_ 590 nm. Samples containing an excess of cyclosulfate (12) were used as positive controls (0% probe labelling), samples without inhibitors for 100% labelling as negative controls and samples without probe as blanks to correct for background polarization.

All samples were corrected for background polarization and the residual enzyme activity was calculated based on the polarization signal from the controls. Polarization signals were plotted against time or inhibitor concentration and processed in GraphPad Prism 9.0. IC_50_ values were calculated *via* non-linear regression using mentioned software (*n* = 2).

### FluoPol-ABPP screen of the iminosugar library

The screen on the iminosugar library, using the optimized conditions as described above, was conducted in 384-well black-bottom plates (Greiner) with final reaction volumes of 15 μL. Final concentration of the iminosugar library compounds was 5 μM. The FluoPol signal was measured on a ClarioStar Plus (BMG Labtech). Resulting polarization signals were processed as described above. Residual enzyme activities were plotted against the corresponding compound ID.

### Analysis of iminosugars as inhibitors of enzymatic activity of rhGAA and GANAB

#### rhGAA

To determine *in vitro* apparent IC_50_ values, 12.5 μL of pure recombinant human enzyme (Myozyme® from Genzyme) at 5 μg mL^−1^ was pre-incubated with 12.5 μL of inhibitor for 30 min at 37 °C in 150 mM McIlvaine buffer pH 4.0 supplemented with 0.1% bovine serum albumin (BSA) (w/v). Subsequently, the reaction mixture was incubated for 30 min with 3.0 mM 4-methylumbeliferone (4MU)-α-d-glucopyranoside (Sigma). The reaction was stopped with excess 1 M NaOH-Glycine (pH 10.3), liberated 4MU fluorescence was measured with a fluorimeter LS55 (Perkin Elmer) using *λ*_Ex_ 366 nm and *λ*_Em_ 445 nm.

#### GANAB

Cellular homogenates of Pompe fibroblasts (1 mg mL^−1^) were used as protein source for GANAB. Similarly, 12.5 μL of homogenate was incubated with 12.5 μL of inhibitor for 30 min at 37 °C and activity was measured with 3.0 mM 4MU-α-d-glucopyranoside in 150 mM McIlvaine pH 7.0 with 0.1% BSA (w/v) after 2 hours incubation. Reactions were stopped with excess 1 M NaOH–glycine (pH 10.3) and measured as described above for GAA.

Observed fluorescence was curve-fitted against inhibitor or substrate concentrations using GraphPad Prism 9.0 in order to obtain the IC_50_ values (Fig. S8†). All IC_50_ values were determined in technical triplicate.

## Data availability

The authors declare that all data supporting the findings of this study are available within the article and ESI,† and raw data files are available from the corresponding author upon request.

## Author contributions

MA synthesised the TAMRA-ABPs I and II, and DvdG executed the FluoPol-ABPP assays and together with MJF and ML performed the fluorogenic substrate assays and ABP labelling under the guidance of MA, HSO, PPG and JMFGA. RJR obtained the crystal structure of rhGBA1 in complex with ABP IV under the guidance of GJD and GS with the assistance of VRZ obtained the crystal structures of rhGAA in complex with *N*-PNT-DNM 15 and ABP II, respectively. DvdG, HSO and MA wrote the manuscript. HSO and MA conceived the research and MA supervised the work.

## Conflicts of interest

The authors declare no competing financial interest.

## Supplementary Material

SC-014-D3SC01021J-s001
