# Supplementary material for: Fluorescence polarisation activity-based protein profiling for the identification of deoxynojirimycin-type inhibitors selective for lysosomal retaining alpha- and beta-glucosidases
Source: Chem Sci. 2023 Aug 8;14(34):9136–44. doi: 10.1039/d3sc01021j (PMC10466331; doi:10.1039/d3sc01021j)
Supplement: SC-014-D3SC01021J-s001 [file SC-014-D3SC01021J-s001.pdf]

Electronic Supplementary Material (ESI) for Chemical Science.
This journal is © The Royal Society of Chemistry 2023

Supporting Information for

Fluorescence Polarisation Activity-Based Protein Profiling in the Identification of Deoxynojirimycin-type Inhibitors selective for Lysosomal Retaining Alpha- and Beta-Glucosidases

Daniël van der Gracht,[a] Rhianna J. Rowland,[b] Véronique Roig-Zamboni,[c] Maria J. Ferraz,[a] Max Louwerse,[a] Paul P. Geurink,[d] Johannes M. F. G. Aerts,[a] Gerlind Sulzenbacher,[c] Gideon J. Davies,[b] Herman S. Overkleeft[a]* and Marta Artola[a]*

[a]Leiden Institute of Chemistry, Leiden University, P. O. Box 9502, 2300 RA Leiden,, The Netherlands

[b]York Structural Biology Laboratory, Department of Chemistry, The University of York, York YO10 5DD, UK.

[c]Architecture et Fonction des Macromolécules Biologiques (AFMB), CNRS, Aix-Marseille University, Marseille, France.

[d]Department of Cell and Chemical Biology, Leiden University Medical Centre, 2333 ZC Leiden, The Netherlands.

Corresponding authors: m.e.artola@lic.leidenuniv.nl; h.s.overkleeft@chem.leidenuniv.nl

TABLE OF CONTENTS

1. Supporting Figures and Tables S3
2. Materials and Methods S42
    2.1. Crystallographic data collection and refinement statistics S42
3. NMR Spectra S45
4. References S49

1. Supporting Figures and Tables

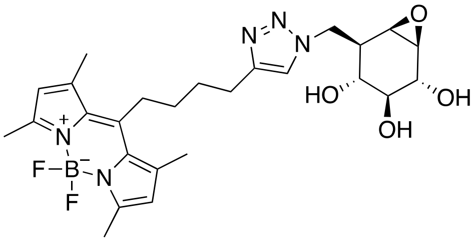

Figure S1. Chemical structure of **ABP IV** (MDW933).

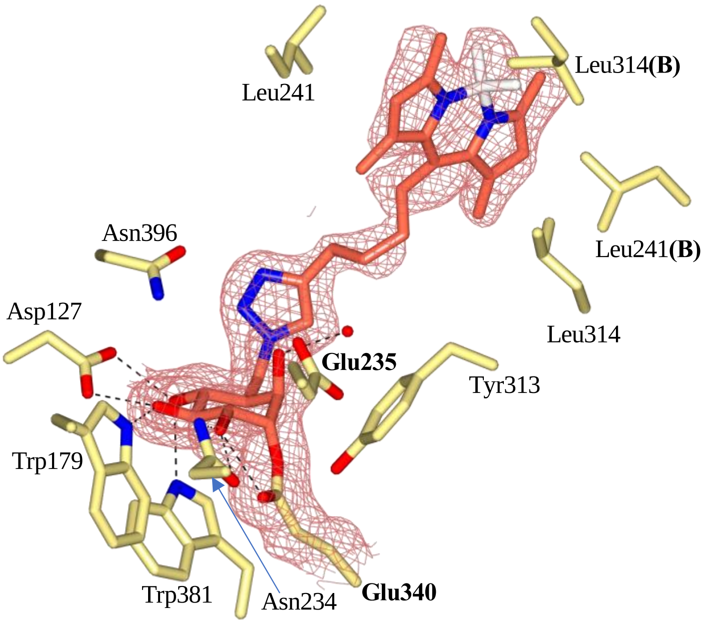

Figure S2. Crystal structure of **ABP IV** in complex with rhGBA1. Electron density for **ABP IV** bound covalently to the catalytic nucleophile (Glu340) Electron density map ($2F_o$-F_c) contoured to 0.7 σ (0.23 e/Å^3) .

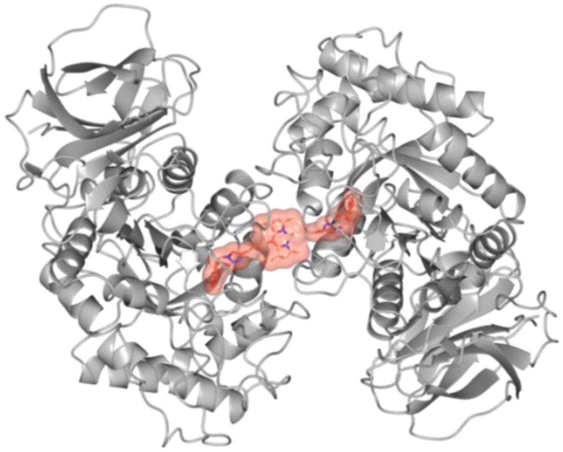

Figure S3. Ribbon diagram of the rhGBA1 dimer with surface depiction of the BODIPY tag of **ABP IV** bound at the dimer interface (red surface).

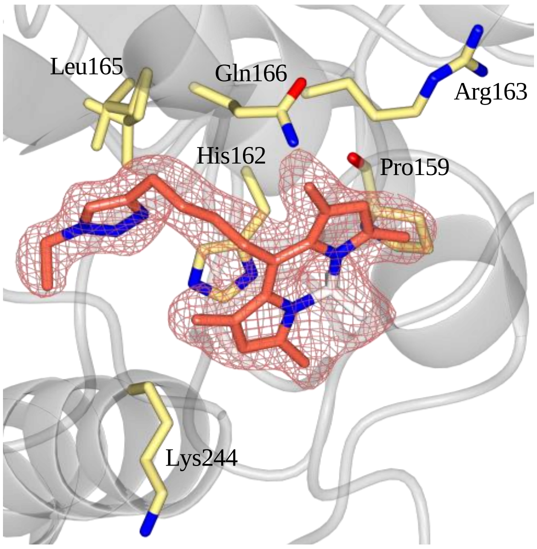

Figure S4. Electron density observed for the BODIPY tag and the triazole-alkyl linker of a decomposed probe bound in a distant site on the surface of the TIM-barrel domain of rhGBA1. The BODIPY tag of **ABP IV** appears to stack with His162. Electron density map (2F_o-F_c) contoured to 1 σ (0.33 e/Å^3).

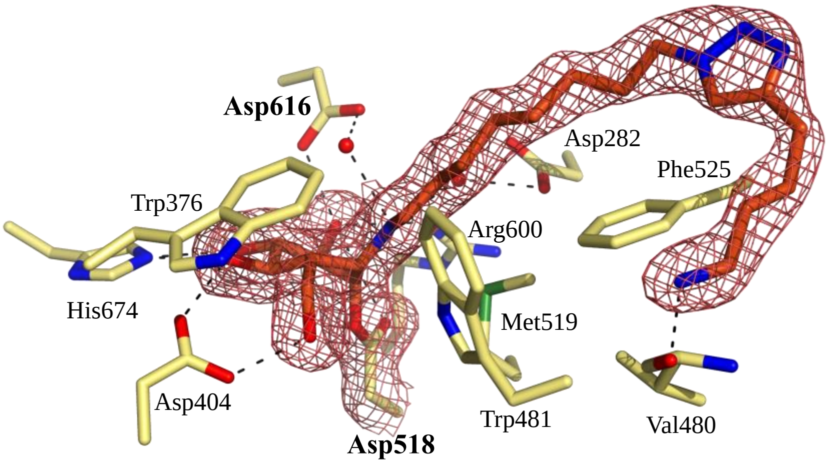

Figure S5. Crystal structure of rhGAA in complex with **ABP II**. The probe has reacted covalently with the nucleophile Asp518 and adopts a $^1S_3$ conformation. Electron density map (2F_o-F_c) contoured at 1.0 σ (0.07 e/Å^3).

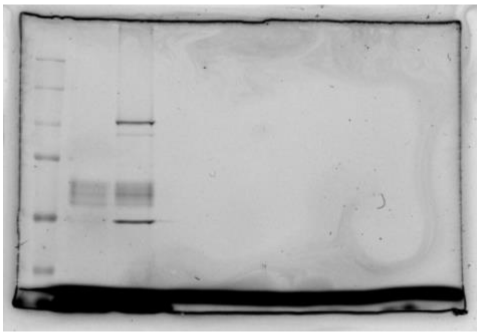

Figure S6. Selective GBA1 labelling by ABP **I** and labeling of GBA1, GBA2 and GBA3 by broad spectrum ABP **III** (Fig. 3d).

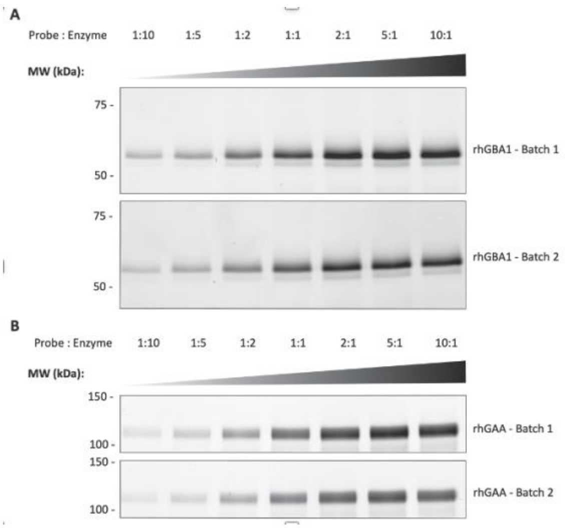

Figure S7. Labelling of rhGBA1 (A) and rhGAA (B) by ABP **I** and **II**, respectively, at different probe:enzyme concentration ratios.

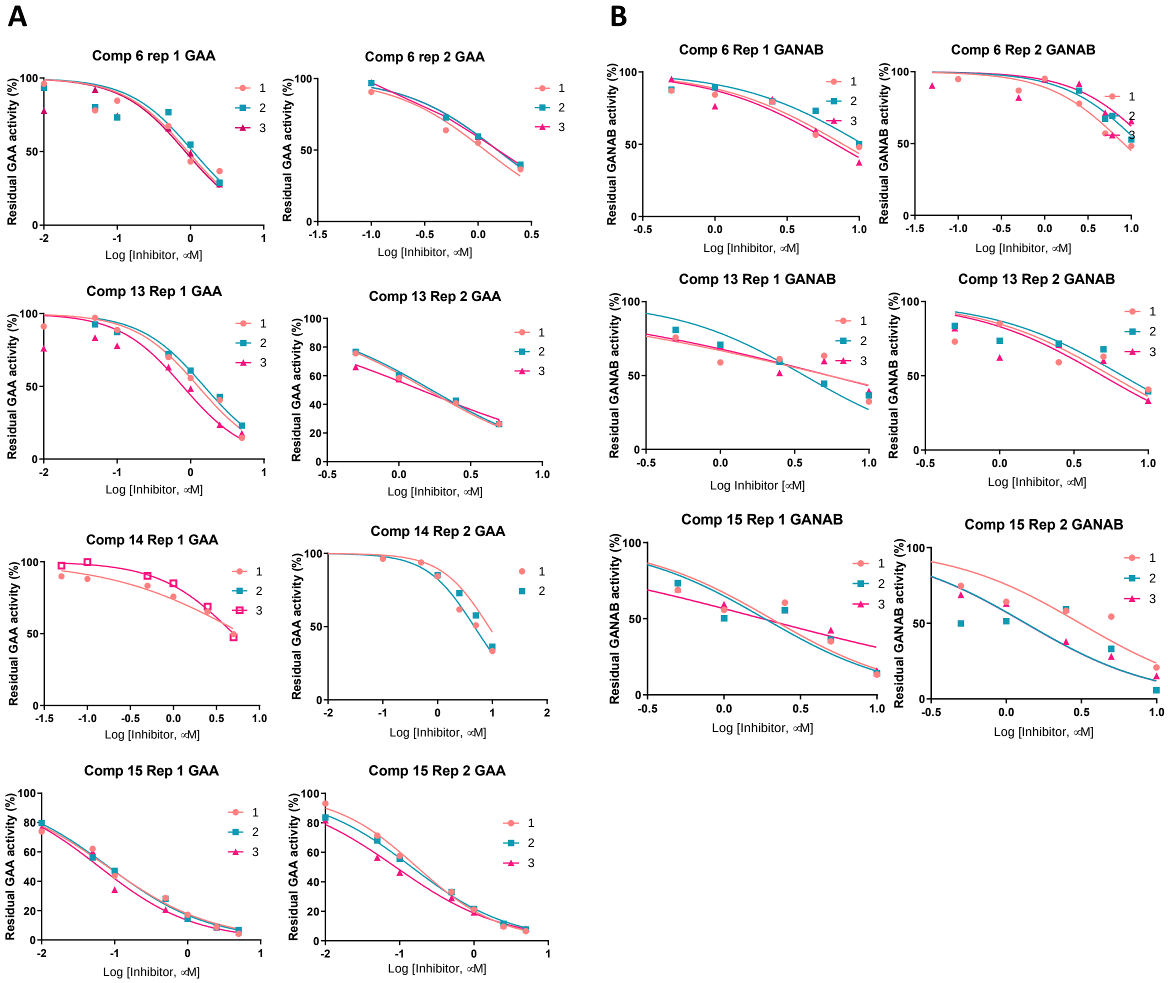

Figure S8. Inhibition curves using 4-MU alpha glucoside as fluorogenic substrate. Two replicates are shown for compounds **6** and **13**-**15** in rhGAA (A) and GANAB (B) where residual enzyme activity (%) *versus* the logarithm of different concentrations of inhibitors is plotted (See Table 1 for IC_{50} values).

Table S1. Chemical structures of the 358 compounds of the Leiden iminosugar library which has been screened in the GBA1 and GAA FluoPol ABPP assays.

**ID#**<br><br>**Compound number in manuscript**	**Structure**	**Ref**
1#	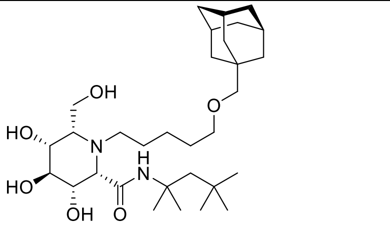	1
2#	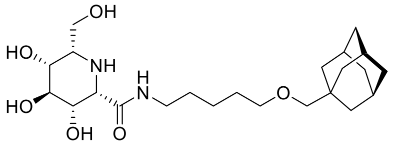	1
3#	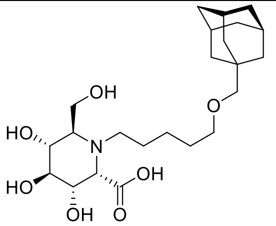	1
4#	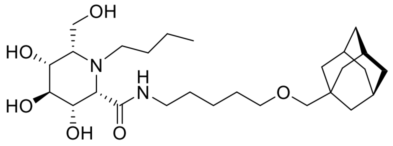	1
5#	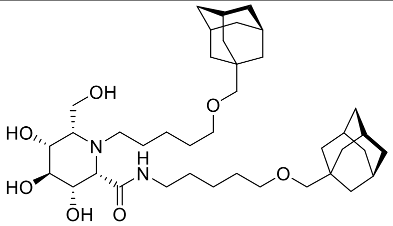	1
6#	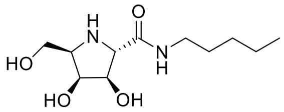	2
7#	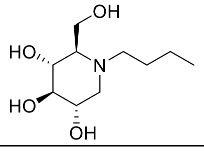	3

8#	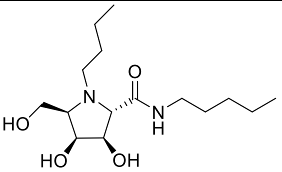	2
9#	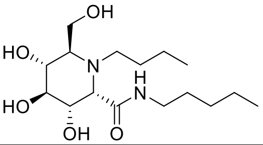	1
10#	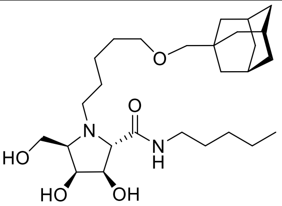	2
11#	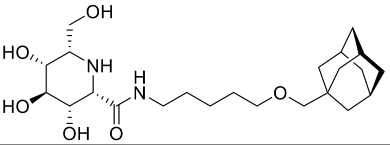	1
12#	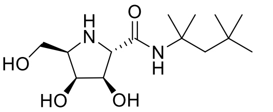	2
13#	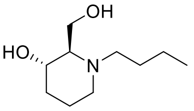	4
14#	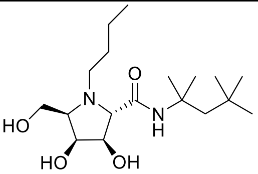	2
15#	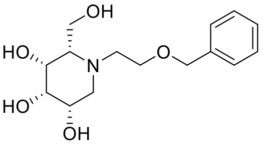	5
16#	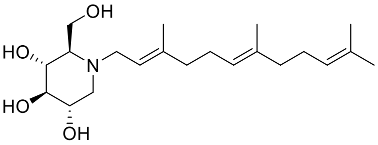	5
17#	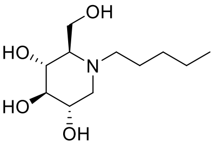	3
18#	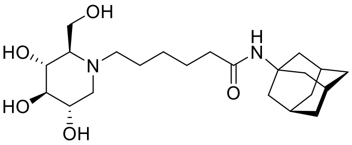	6
19#	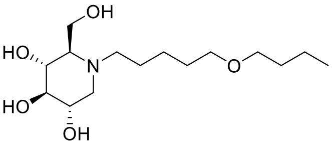	3

20#	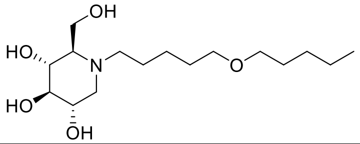	3
21#	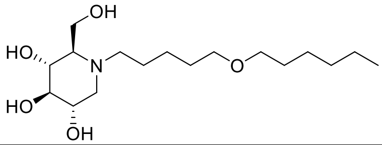	3
22#	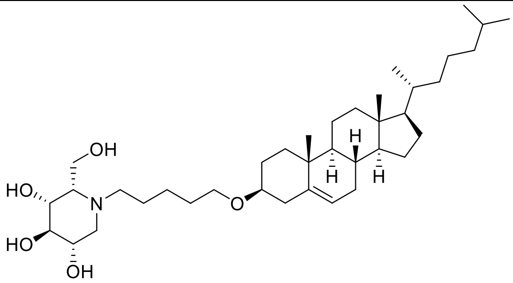	6
23#	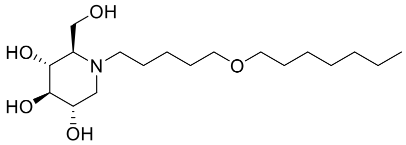	3
24#	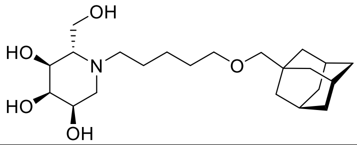	5
25#	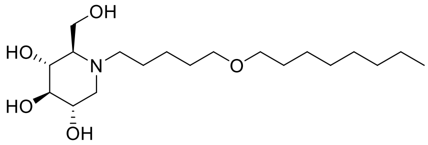	3
26#	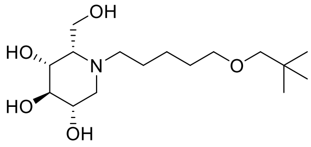	5
27#	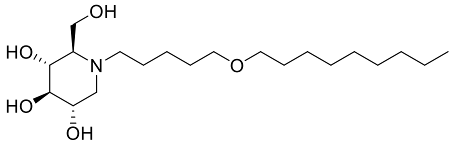	3
28#	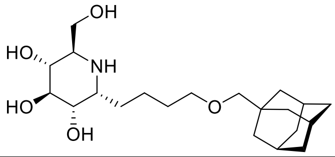	7
29#	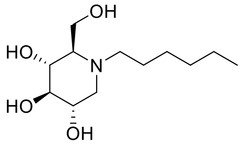	3
30#	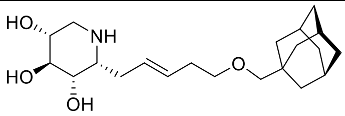	7

31#	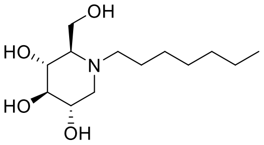	3
32#	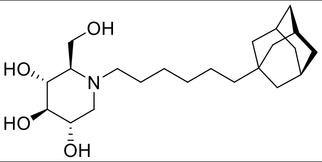	6
33#	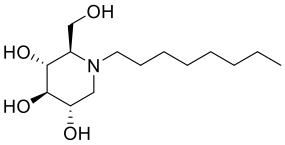	3
34#	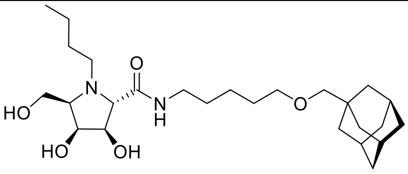	2
35#	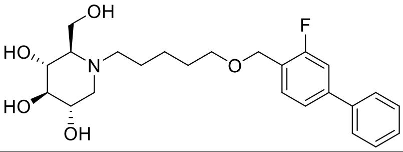	6
36#	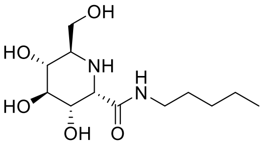	1
37#	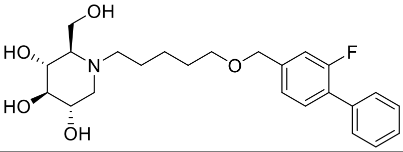	6
38#	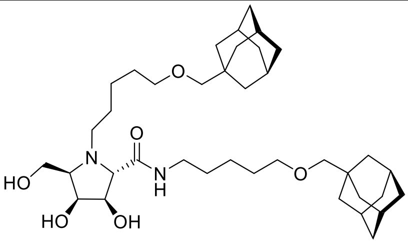	2
39#	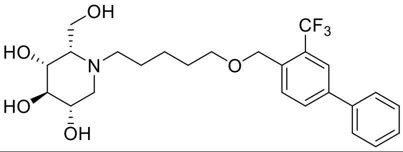	6
40#	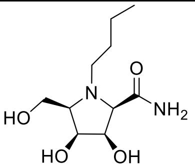	2
41#	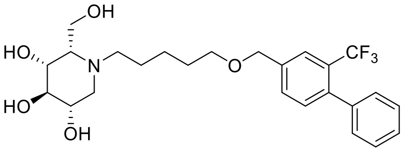	6

42#	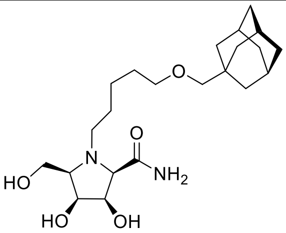	2
43#	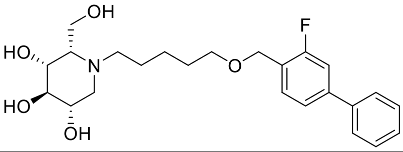	6
44#	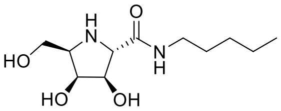	2
45#	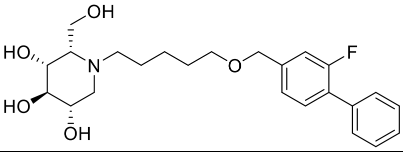	6
46#	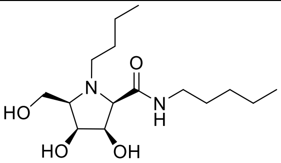	2
47#	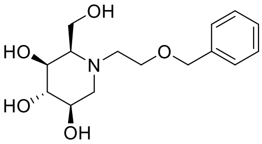	5
48#	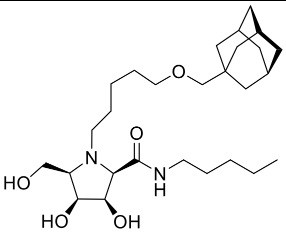	2
49#	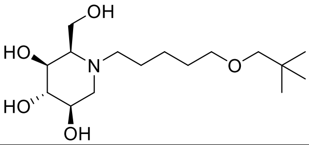	5
50#	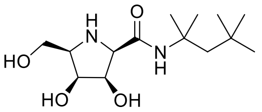	2
51#	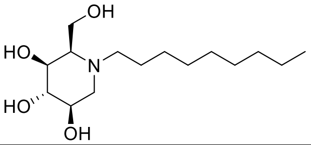	5
52#	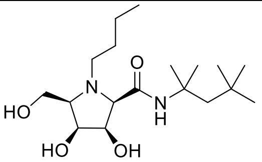	2

53#	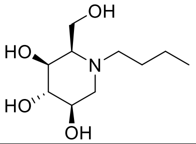	5
54#	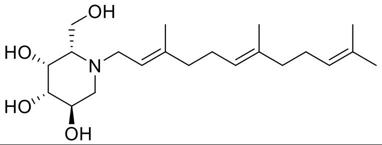	5
55#	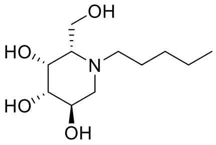	3
56#	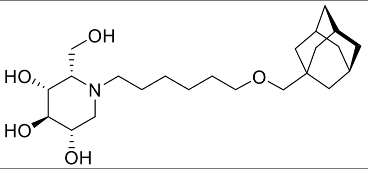	8
57#	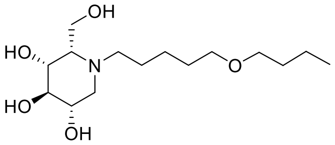	3
58#	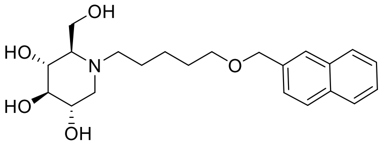	6
59#	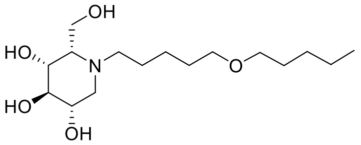	3
60#	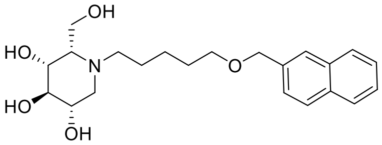	6
61#	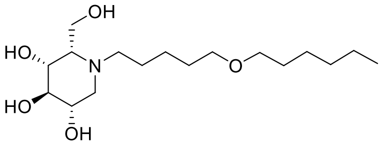	3
62# **Compound 6**	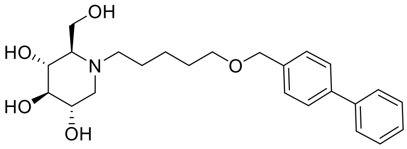	6
63#	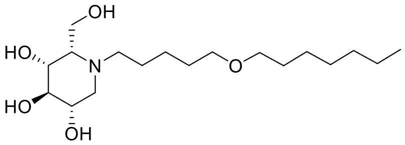	3
64#	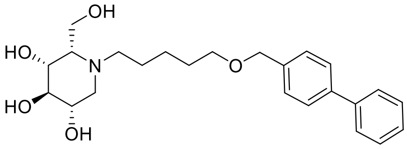	6

65#	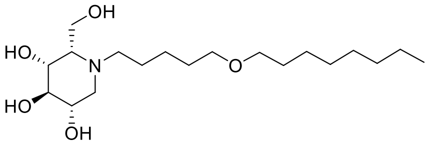	3
66#<br><br>**Compound 15**	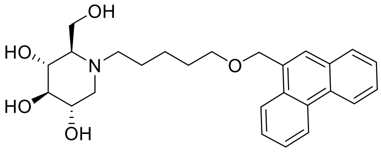	6
67#	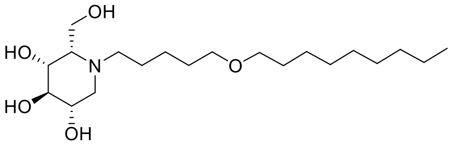	3
68#	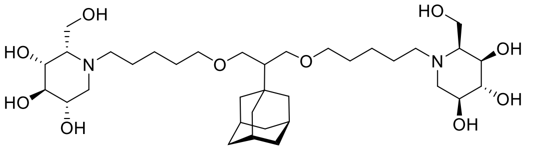	9
69#	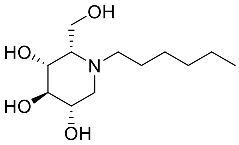	3
70#	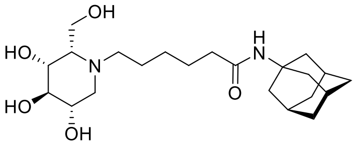	6
71#	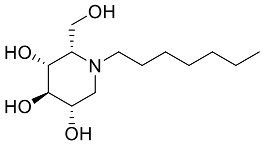	3
72#	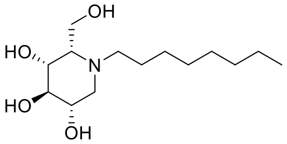	3
73#	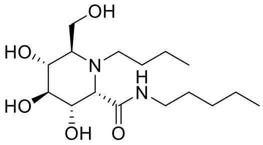	1
74#	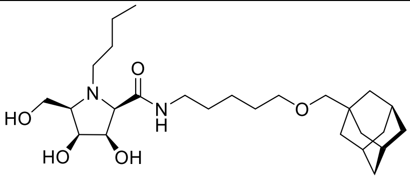	2
75#	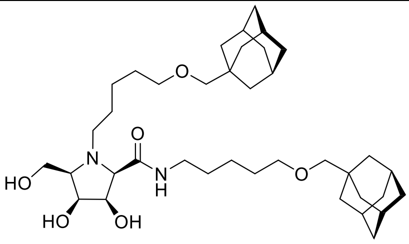	2

76#	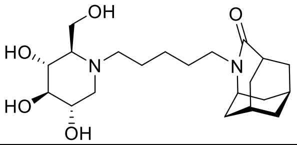	5
77#	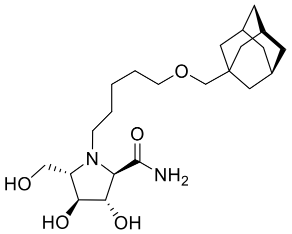	2
78#	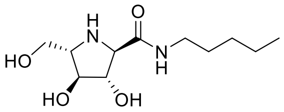	2
79#	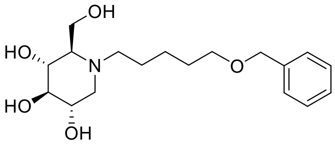	6
80#	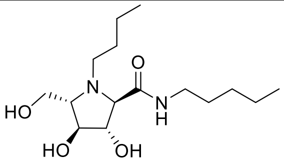	2
81#	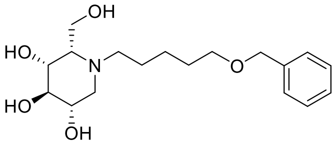	6
82#	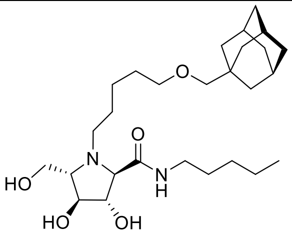	2
83#	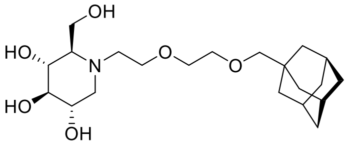	5
84#	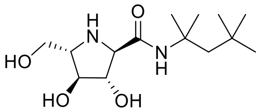	2
85#	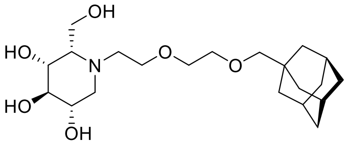	5
86#	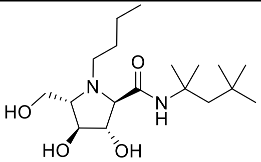	2

87#	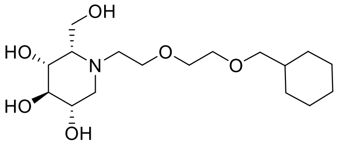	5
88#	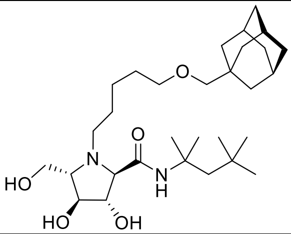	2
89#	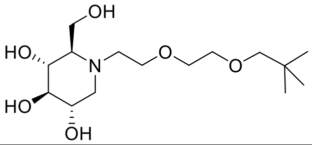	5
90#	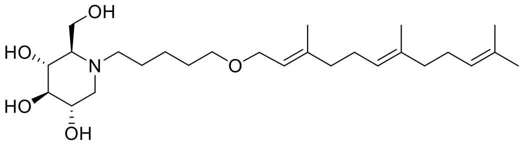	6
91#	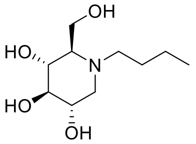	3
92#	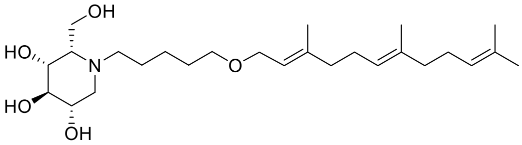	6
93#	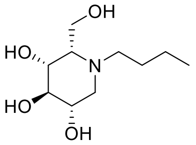	3
94#	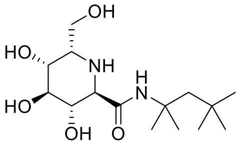	1
95#	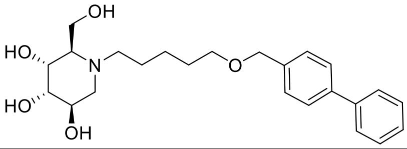	5
96#	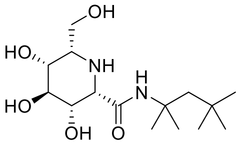	1

97#<br><br>**Compound 14**	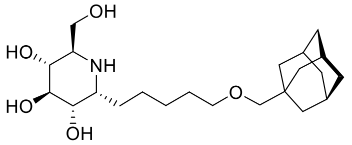	7
98#	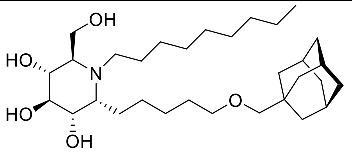	7
99#	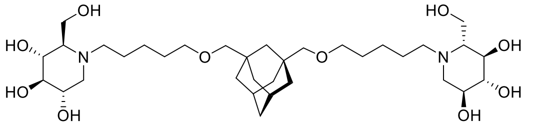	9
100#	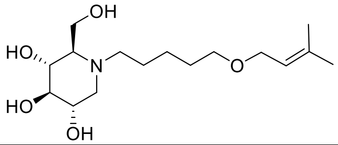	5
101#	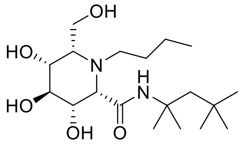	1
102#	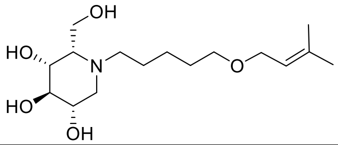	5
103#	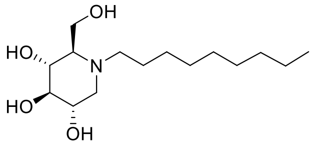	3
104#	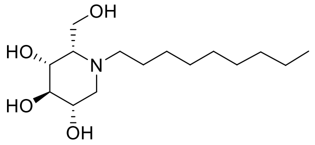	3
105#	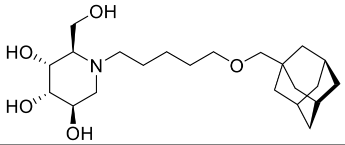	5
106#	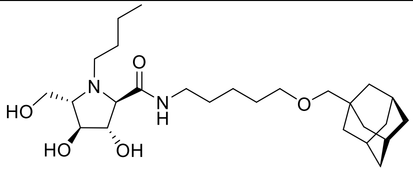	2
107#	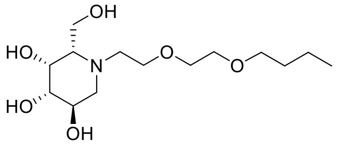	5

108#	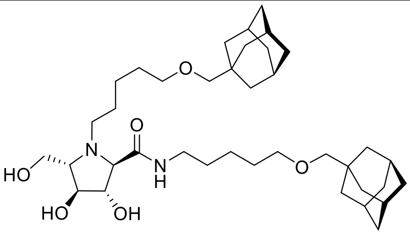	2
109#	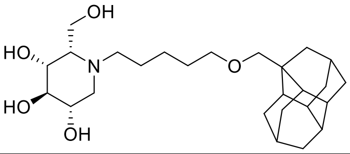	5
110#	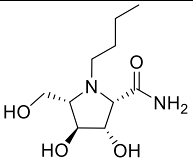	2
111#	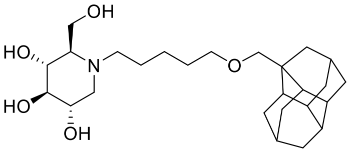	5
112#	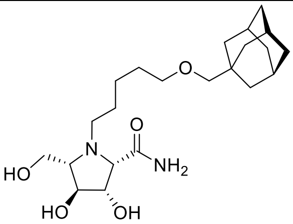	2
113#<br><br>**Compound 13**	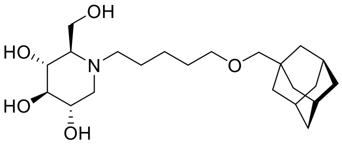	10
114#	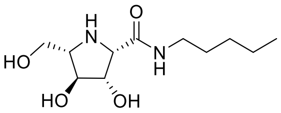	2
115#	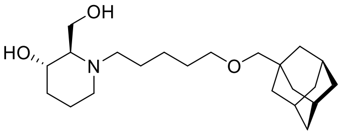	4
116#	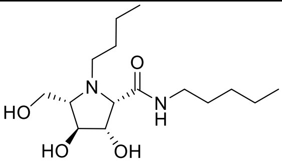	2
117#	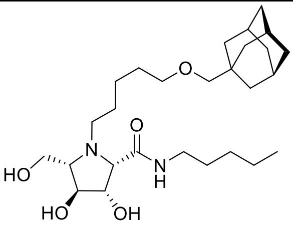	2

118#	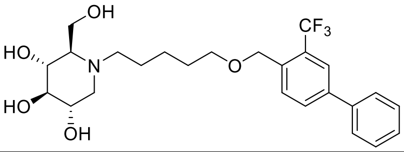	6
119#	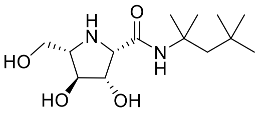	2
120#	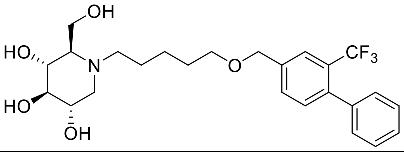	6
121#	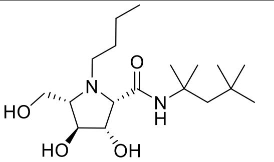	2
122#	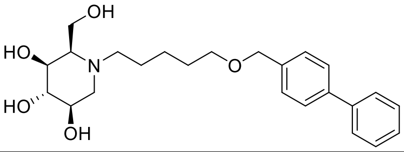	6
123#	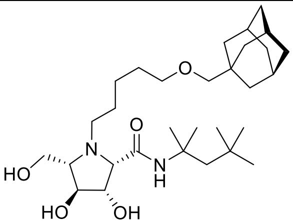	2
124#	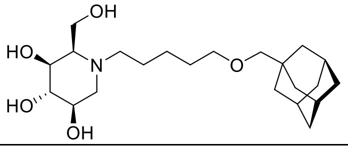	5
125#	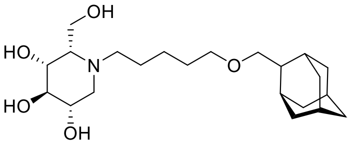	6
126#	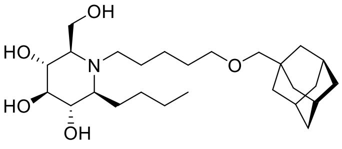	7
127#	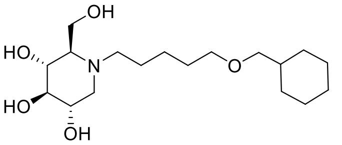	6
128#	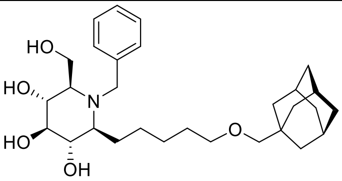	7

129#	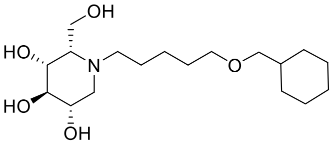	6
130#	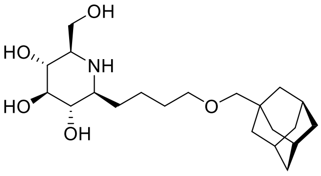	7
131#	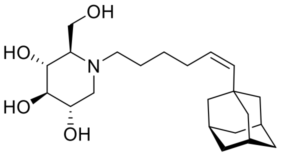	6
132#	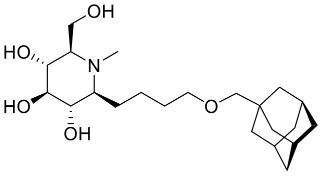	7
133#	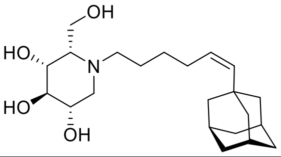	6
134#	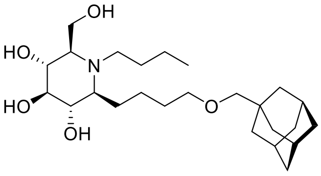	7
135#	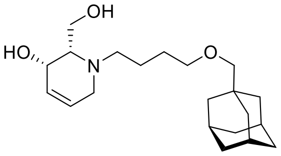	4
136#	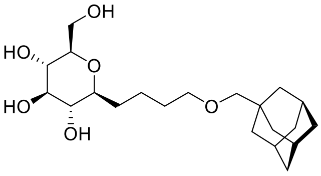	7
137#	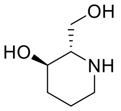	4
138#	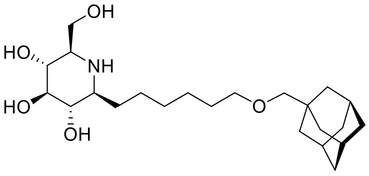	7
139#	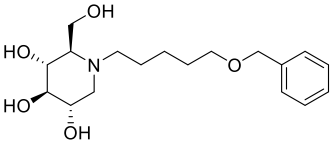	6

140#	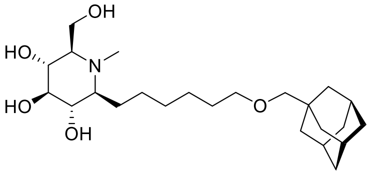	7
141#	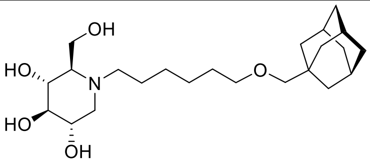	6
142#	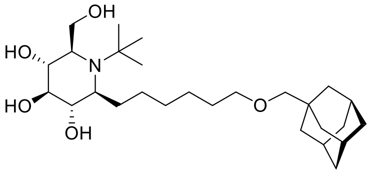	7
143#	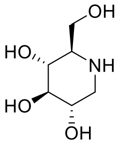	8
144#	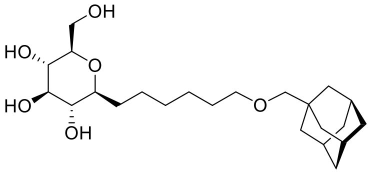	7
145#	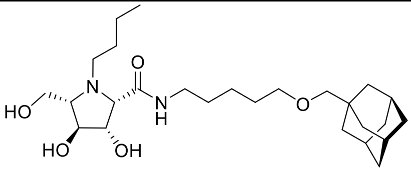	2
146#	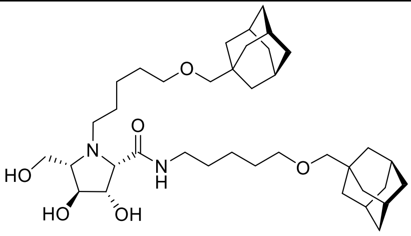	2
147#	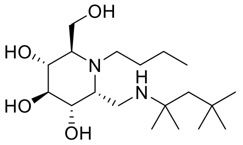	7
148#	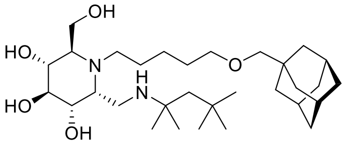	7
149#	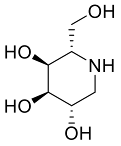	8

150#	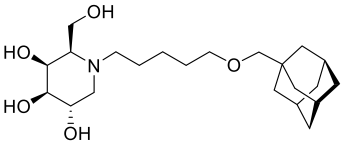	8
151#	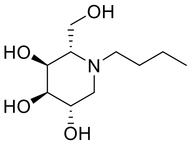	8
152#	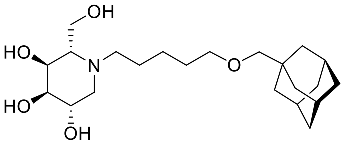	8
153#	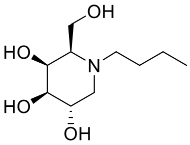	8
154#	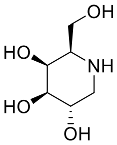	8
155#	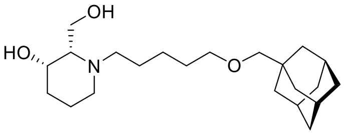	4
156#	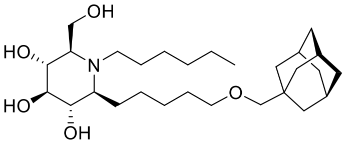	7
157#	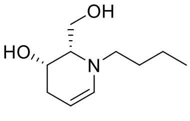	4
158# **Compound 8**	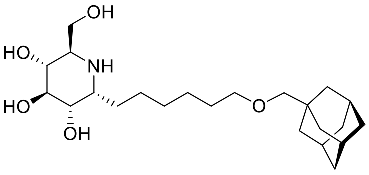	7
159#	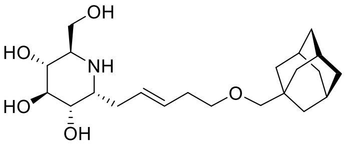	7

160#	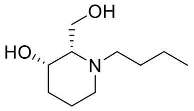	4
161#	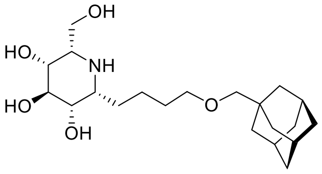	7
162#	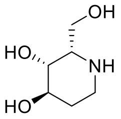	4
163#	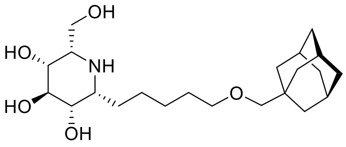	7
164#	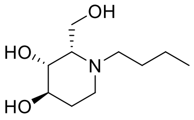	4
165#	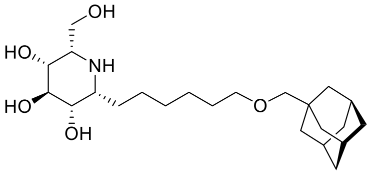	7
166#	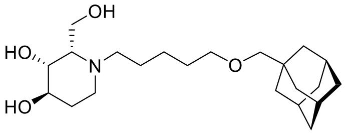	4
167#	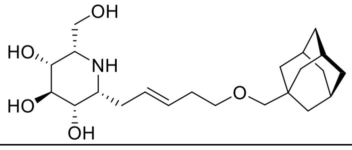	7
168#	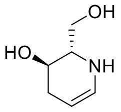	4
169#	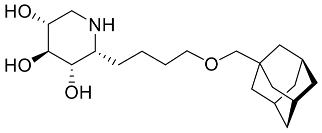	7
170#	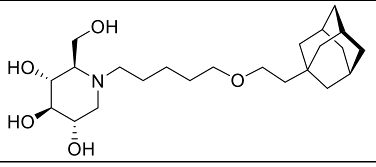	5

171#	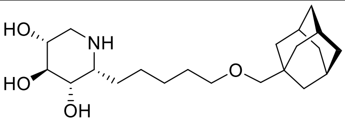	7
172#<br><br>**Compound 7**	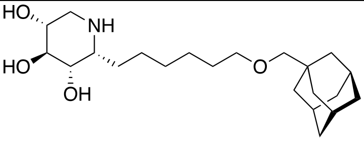	7
173#	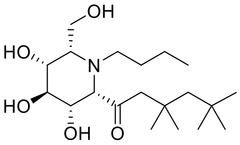	1
174#	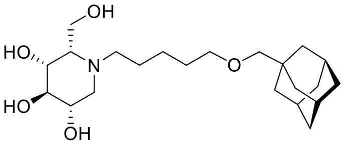	8
175#	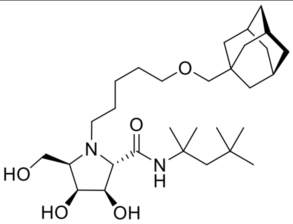	2
176#	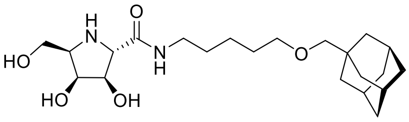	2
177#	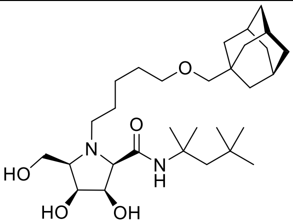	2
178#	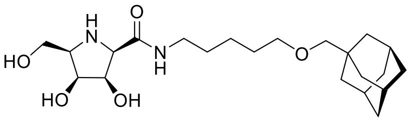	2
179#	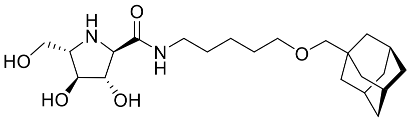	2
180#	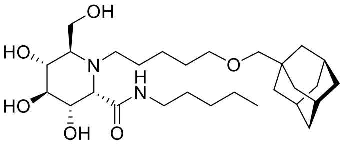	1
181#	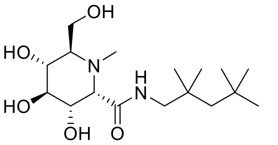	1

182#	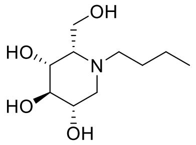	8
183#	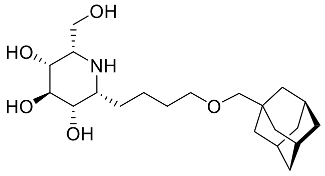	7
184#	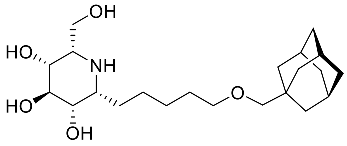	7
185#	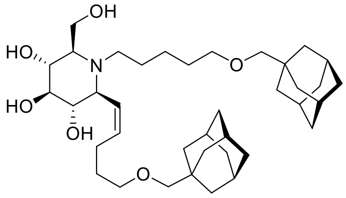	7
186#	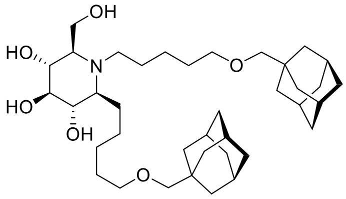	7
187#	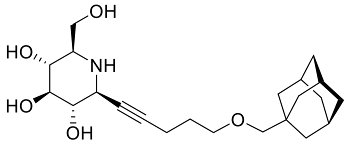	7
188#	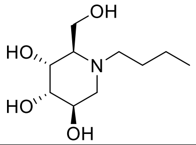	5
189#	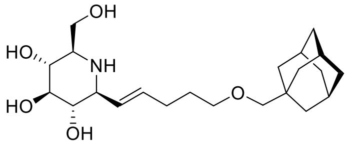	7
190#	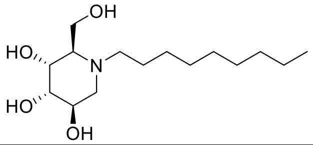	5
191#	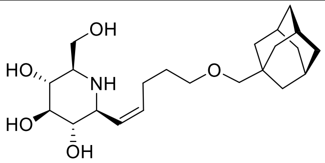	7
192#	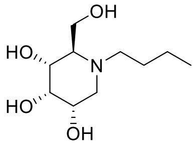	5

193#	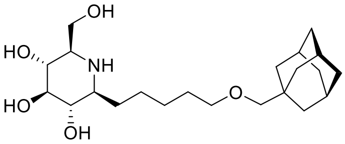	7
194#	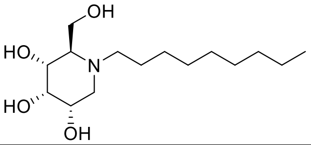	5
195#	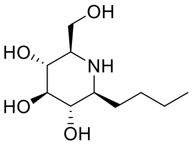	7
196#	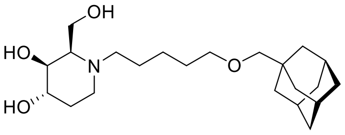	4
197#	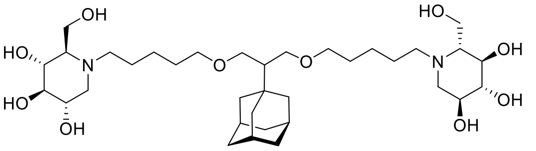	9
198#	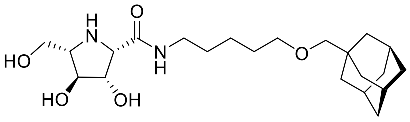	2
199#	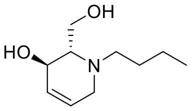	4
200#	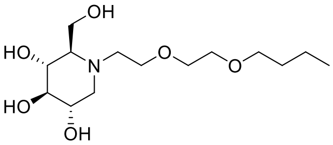	5
201#	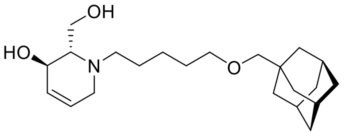	4
202#	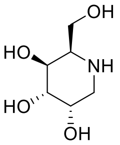	11
203#	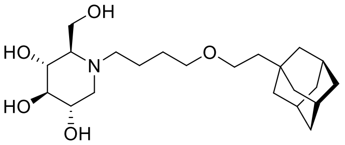	5
204#	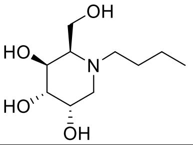	5

205#	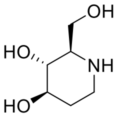	4
206#	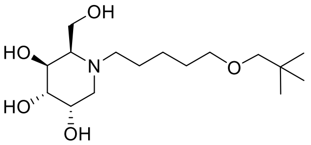	5
207#	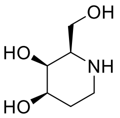	4
208#	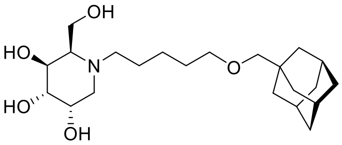	5
209#	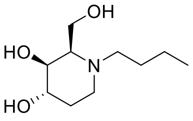	4
210#	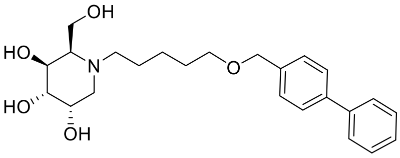	5
211#	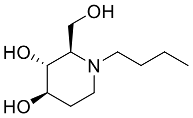	4
212#	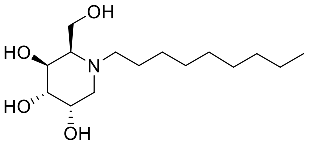	5
213#	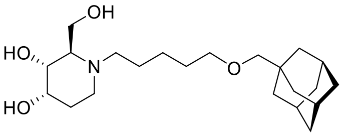	4
214#	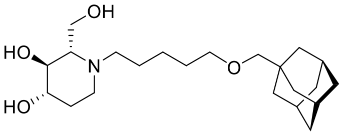	4
215#	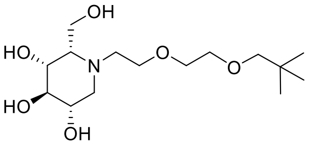	5

216#	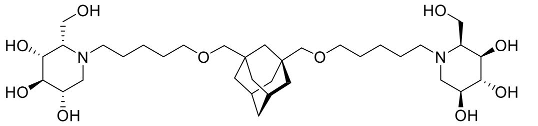	9
217#	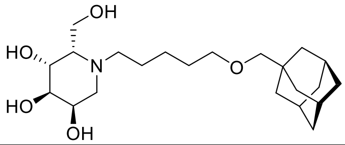	5
218#	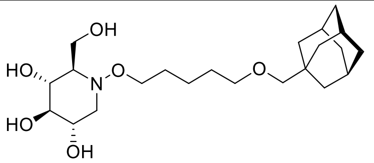	5
219#	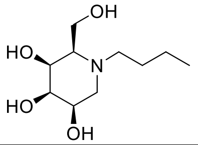	5
220#	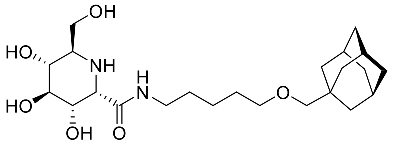	1
221#	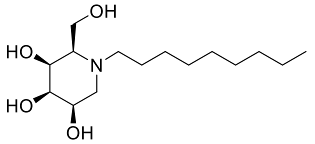	5
222#	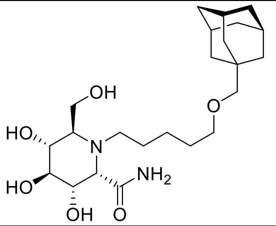	1
223#	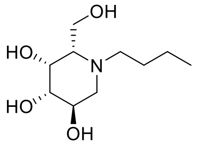	5
224#	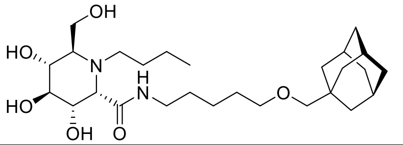	1
225#	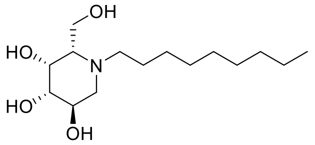	5
226#	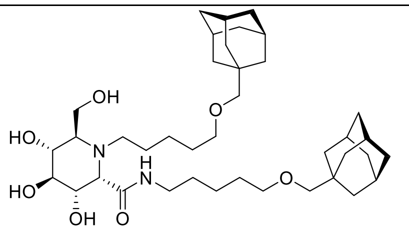	1

227#	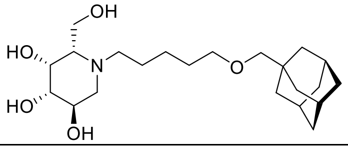	5
228#	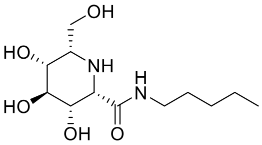	1
229#	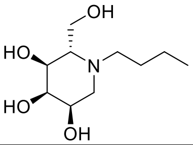	5
230#	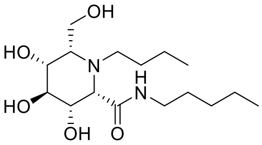	1
231#	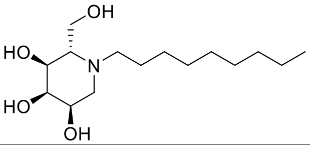	5
232#	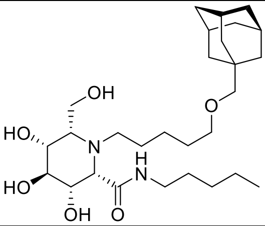	1
233#	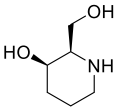	4
234#	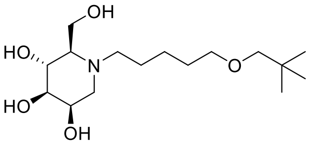	5
235#	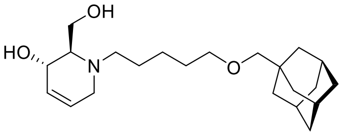	4
236#	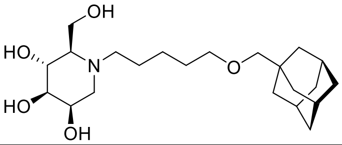	5
237#	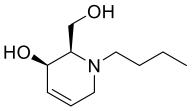	4

238#	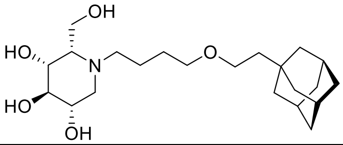	5
239#	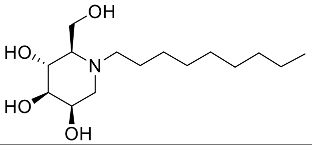	5
240#	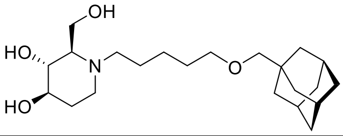	4
241#	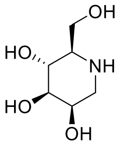	5
242#	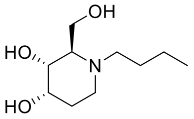	4
243#	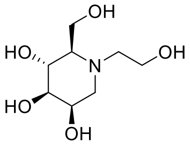	5
244#	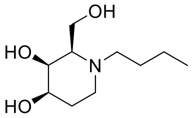	4
245#	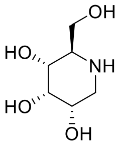	5
246#	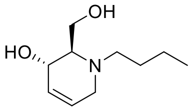	4
247#	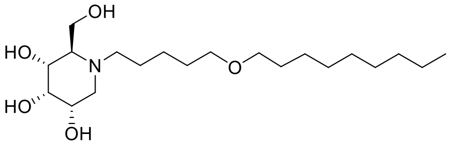	5
248#	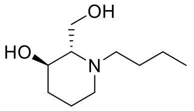	4
249#	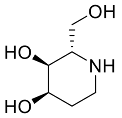	4
250#	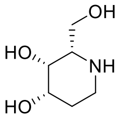	4

251#	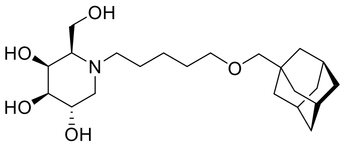	8
252#	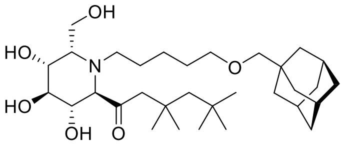	1
253#	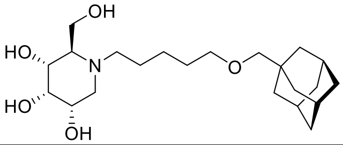	5
254#	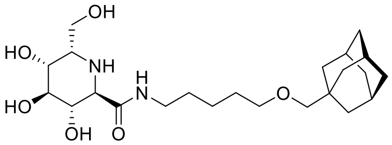	1
255#	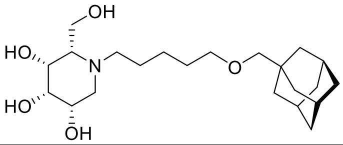	5
256#	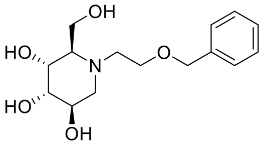	5
257#	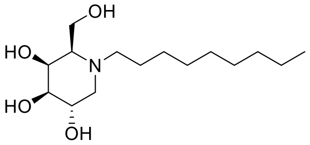	3
258#	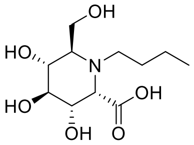	1
259#	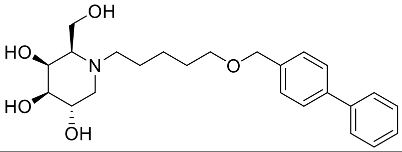	6
260#	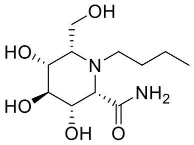	1
261#	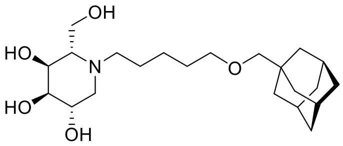	8
262#	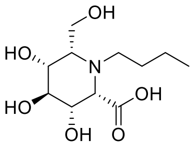	1

263#	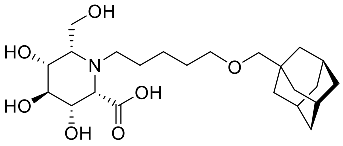	1
264#	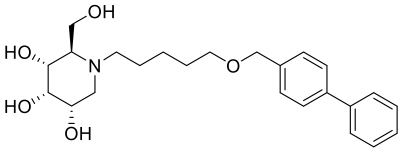	5
265#	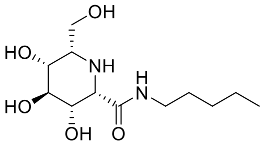	1
266#	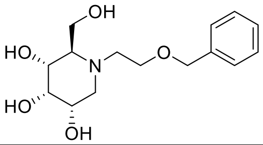	5
267#	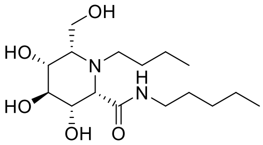	1
268#	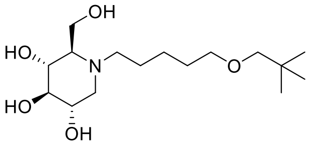	5
269#	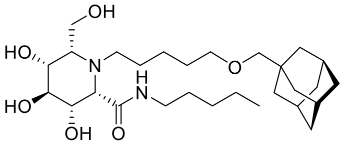	1
270#	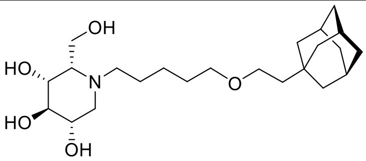	8
271#	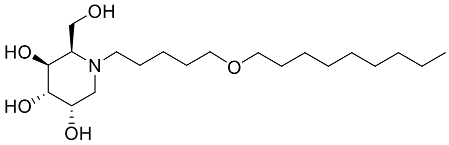	5
272#	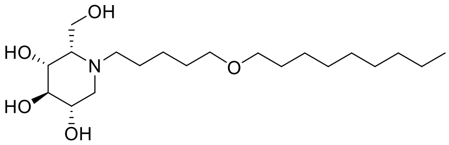	3
273#	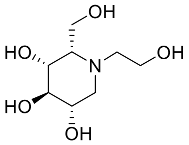	12
274#	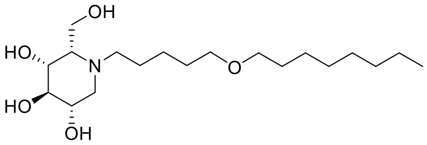	3

275#	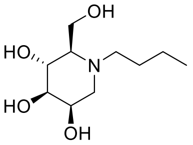	13
276#	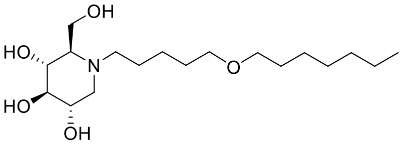	3
277#	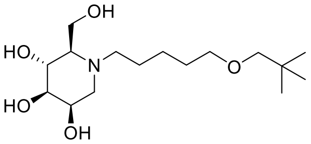	5
278#	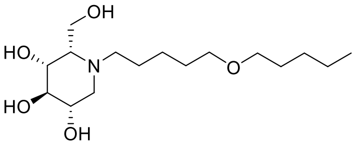	3
279#	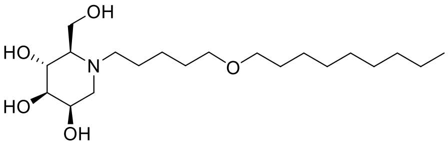	5
280#	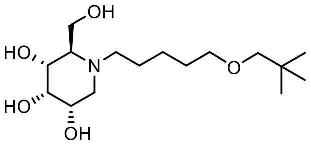	5
281#	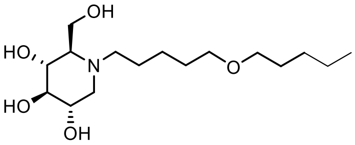	3
282#	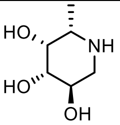	14
283#	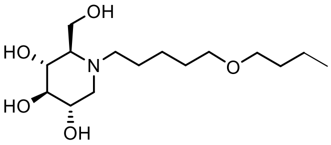	3
284#	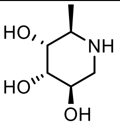	14
285#	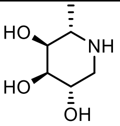	14
286#	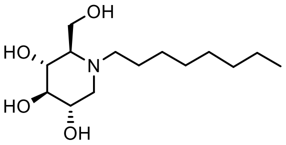	3

287#	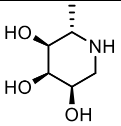	14
288#	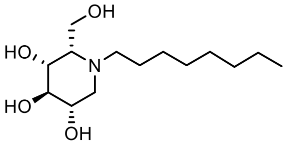	3
289#	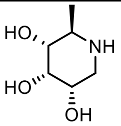	14
290#	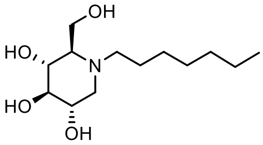	3
291#	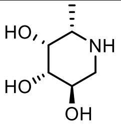	14
292#	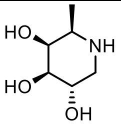	14
293#	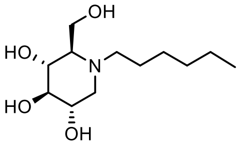	3
294#	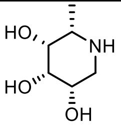	14
295#	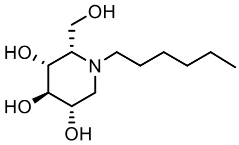	3
296#	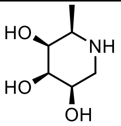	14
297#	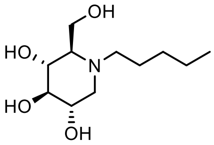	3
298#	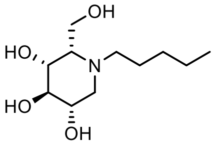	3

299#	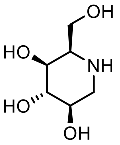	11
300#	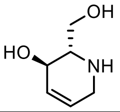	4
301#	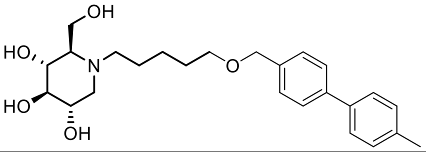	6
302#	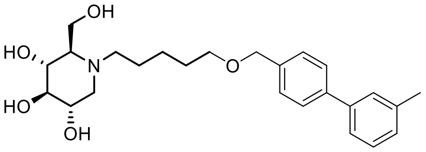	6
303#	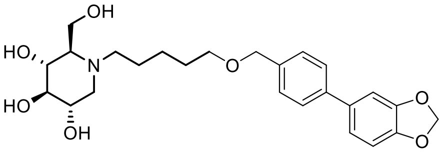	6
304#	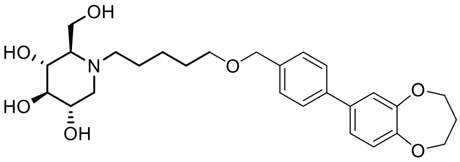	6
305#	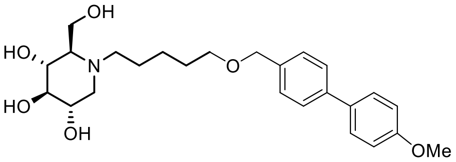	6
306#	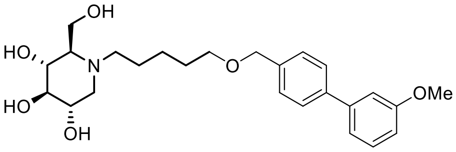	6
307#	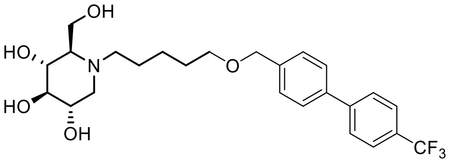	6
308#	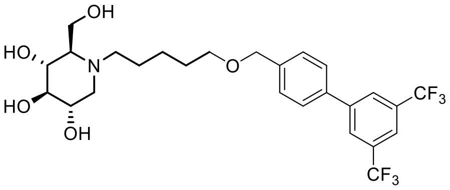	6
309#	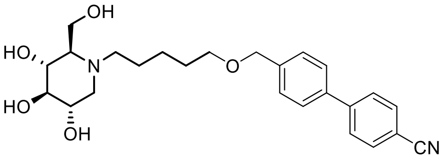	6

310#	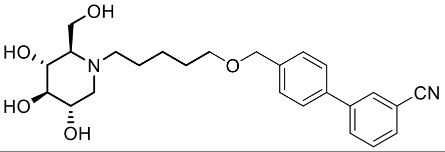	6
311#	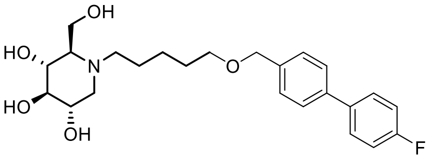	6
312#	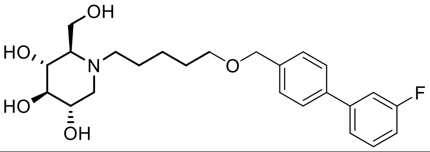	6
313#	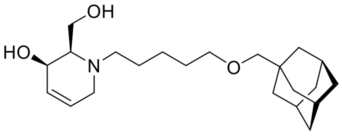	4
314#	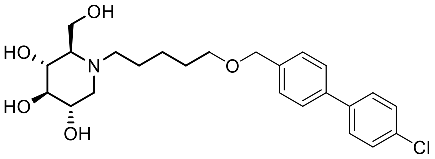	6
315#	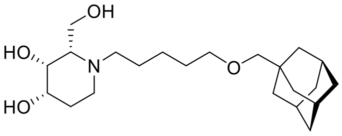	4
316#	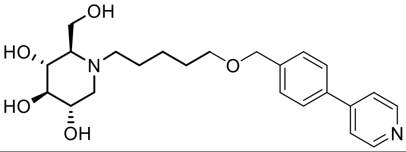	6
317#	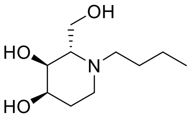	4
318#	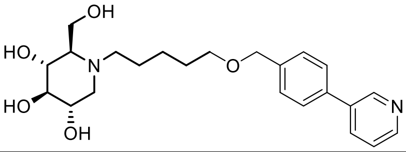	6
319#	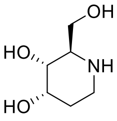	4
320#	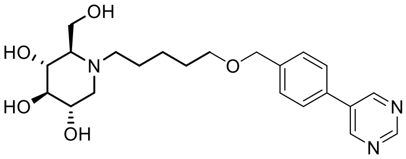	6
321#	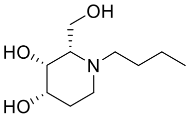	4

322#	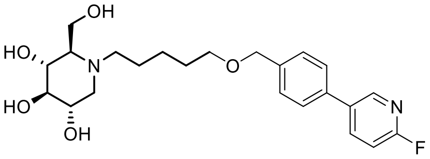	6
323#	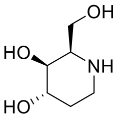	4
324#	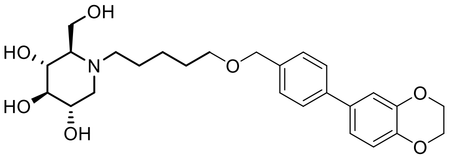	6
325#	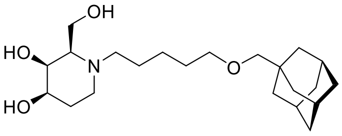	4
326#	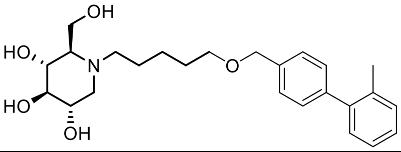	6
327#	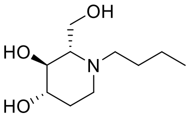	4
328#	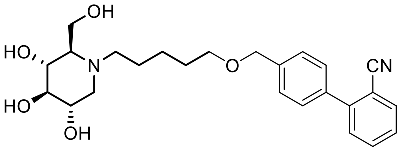	6
329#	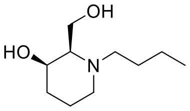	4
330#	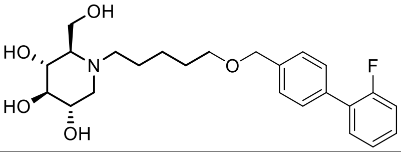	6
331#	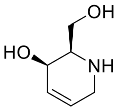	4
332#	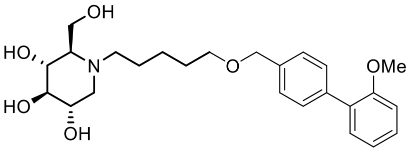	6

333#	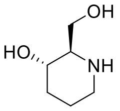	4
334#	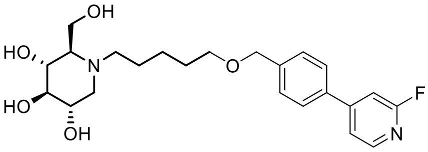	6
335#	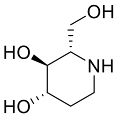	4
336#	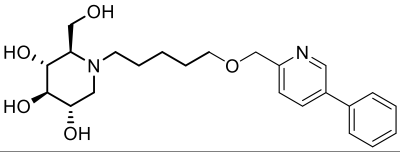	6
337#	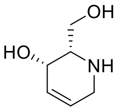	4
338#	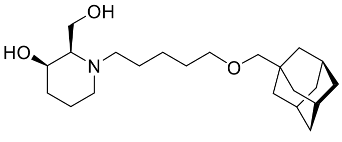	4
339#	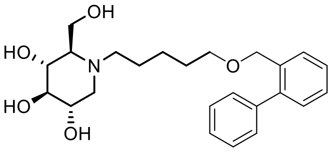	6
340#	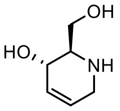	4
341#	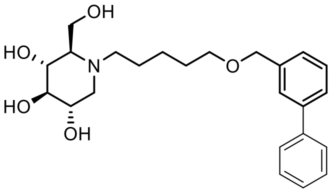	6
342#	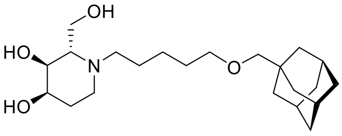	4
343#	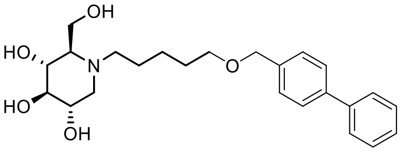	6
344#	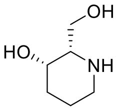	4

345#	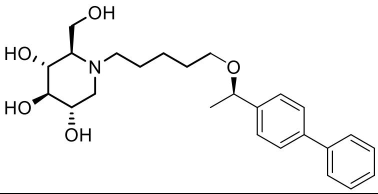	6
346#	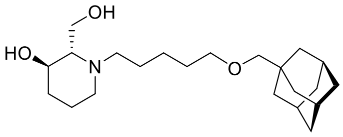	4
347#	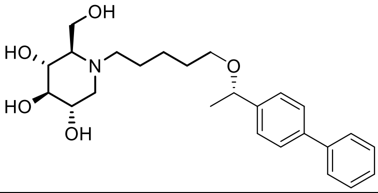	6
348#	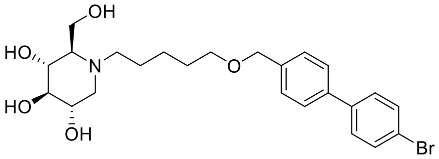	6
349#	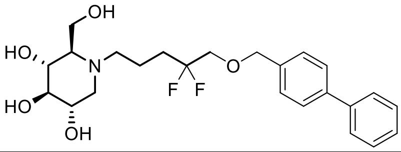	6
350#	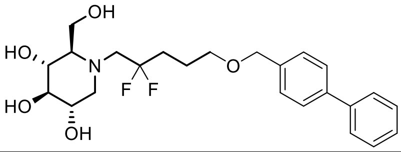	6
351#	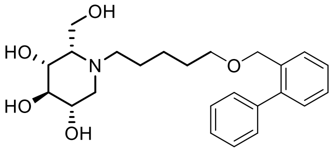	6
352#	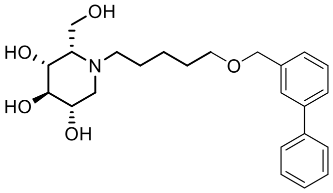	6
353#	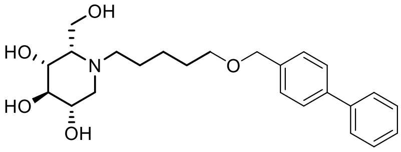	6
354#	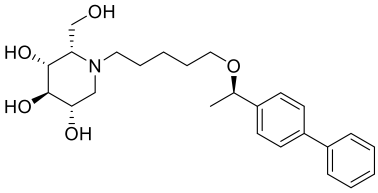	6

355#	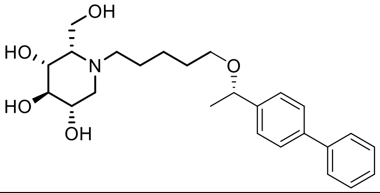	6
356#	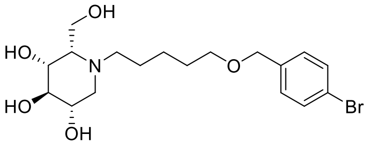	6
357#	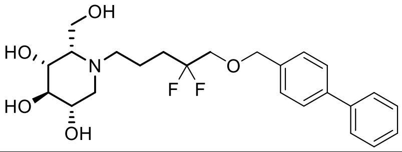	6
358#	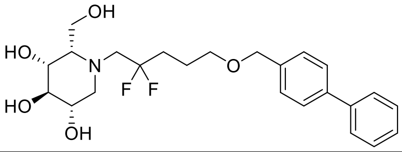	6

Table S2. Iminosugars identified in the rhGAA FluoPol-ABPP assay. Residual FP-signal (%). IC_{50} values for *in vitro* inhibition of lysosomal rhGAA (Myozyme) and ER-α-glucosidase II (GANAB) using 4-MU alpha glucoside substrate.

Cmp	#ID	GAA FP%	IC_{50} on rhGAA (in µM)	IC_{50} on GANAB (in µM)
	**16**	0.9	0.32	8.8
	**18**	24	0.36	2.4
	**19**	28	0.35	3.6
	**20**	17	0.33	19.0
	**21**	17	0.31	14.8
	**22**	6.5	0.17	6.0
	**23**	16	0.29	7.2
	**25**	13	0.10	4.1
	**26**	23	0.64	29
	**27**	8.4	0.28	2.8
	**29**	49	1.5	100
	**31**	35	0.91	60
	**32**	0.3	0.20	1.2
	**33**	28	0.54	16
	**35**	4.8	0.021	0.71
	**37**	3.6	0.037	1.6
	**47**	34	0.55	4.6
	**58**	7.7	0.25	0.85
**6**	**62**	3.8	1.2	8.3
**15**	**66**	25	0.11	2.0
	**89**	24	0.73	1.7

	**90**	6.8	0.49	2.5
14	**97**	34	4.0	>100
	**99**	5.6	0.28	1.3
	**100**	19	0.60	9.9
	**103**	18	0.32	9.0
	**111**	3.2	0.11	0.89
13	**113**	8.9	1.4	5.4
	**118**	2.9	0.018	0.48
	**120**	2.4	0.016	0.72
	**122**	35	1.3	100
	**127**	9.3	0.29	1.84
	**131**	14	0.026	0.44
	**139**	20	0.76	9.32
	**141**	1.8	0.29	0.58
	**150**	15	1.4	12
	**153**	14	3.6	19
	**154**	2.4	8.8	21
8	**158**	29	2.462	100
	**159**	23	0.45	100
	**170**	4.1	0.43	1.6
	**197**	0.4	0.22	1.3
	**200**	46	3.9	100
	**203**	9.2	0.042	2.1
	**276**	0.6	0.072	1.81
	**281**	4.2	0.54	3.7
	**283**	4.4	0.72	4.9
	**286**	9.1	0.76	8.5
	**293**	17	1.3	14
	**297**	12	1.7	100
	**301**	0.5	0.037	1.6
	**302**	0.1	0.020	1.6
	**303**	0.1	0.14	4.7
	**304**	0.6	0.037	1.2
	**305**	0.3	0.020	0.38
	**306**	0.8	0.015	0.39
	**307**	2.5	0.036	1.7
	**308**	1.5	2.4	14
	**309**	0.1	0.037	0.42
	**310**	2.5	0.054	1.0
	**311**	0.9	0.043	1.4
	**312**	0.9	0.024	1.0
	**314**	1.7	0.093	0.15
	**316**	1.1	0.089	1.3
	**318**	0.6	0.085	1.7
	**320**	1.0	0.082	1.3
	**322**	0.8	0.080	1.7
	**324**	0.9	0.012	0.57

	**326**	0.8	0.012	0.69
	**328**	0.7	0.021	1.7
	**330**	0.8	0.098	1.6
	**332**	0.8	0.13	1.3
	**334**	1.5	0.16	1.6
	**336**	0.4	0.037	0.31
	**339**	0.9	0.10	0.62
	**341**	1.8	0.054	0.52
	**343**	0.6	0.043	0.25
	**345**	1.5	0.058	0.37
	**347**	0.4	0.042	0.40
	**348**	3.1	0.023	0.61
	**349**	3.0	0.028	4.4

Table S3. Crystallographic data collection and refinement statistics.

	15 in rhGAA	ABP II in rhGAA	ABP-IV in rhGBA1
**Data collection**			
Space group	$P2_12_12_1$	$P2_12_12_1$	$P2_1$
Cell dimensions			
a, *b*, *c* (Å)	96.75, 102.60, 129.18	97.34, 102.94, 129.92	52.96, 158.42, 68.24
α, β, γ, (°)	90, 90, 90	90, 90, 90	90, 102, 90
Resolution (Å)	48.38-1.75 (1.78-1.75)	48.67-1.90 (1.93-1.90)	79.22-1.86 (1.89-1.86)
R_{merge}	0.120 (2.065)	0.130 (1.675)	0.119 (0.843)
R_{pim}	0.031 (0.537)	0.036 (0.465)	0.049 (0.345)
$CC_{1/2}$	0.999 (0.573)	0.999 (0.695)	0.996 (0.859)
I / σI	14.1 (1.3)	14.5 (1.9)	9.1 (2.0)
Completeness (%)	100 (100)	100 (100)	100 (100)
Redundancy	15.6 (15.6)	13.7 (13.9)	6.9 (7.0)
Wilson B (A^2)	21.0	24.2	22.7
**Refinement**			
Resolution (Å)	47.73-1.75 (1.795-1.75)	48.72-1.90 (1.949-1.90)	66.80-1.86
No. reflections working set	123307 (9022)	98106 (7212)	87211 (6456)
No. reflections test set	6501 (493)	5146 (361)	4665 (329)
R_{work} / R_{free}	0.1422/0.1749	0.1487/0.1779	0.1769/0.2318
No. atoms			
Protein	6661	6691	7819
N-glycans	182	147	120
Ligands/ions	130	134	364
Waters	785	612	744
B-factors (Å^2)			
Protein, main/side chains	39.0/44.2	40.2/45.2	23.8/26.2
N-glycans	74.2	66.8	52.0
Ligands/ions	61.9	63.2	43.7
Waters	52.9	53.6	35.0
R.m.s. deviations			
Bond lengths (Å)	0.010	0.010	0.009
Bond angles (°)	1.62	1.57	1.58
Ramachandran Plot			
Most favourable regions (%)	96.9	97.7	96.5
Allowed regions (%)	3.0	2.2	3.0
PDB	8CB1	8CB6	7NWV

Values in parentheses are for highest resolution shell

2. Materials and Methods

2.1. Crystallographic data collection and refinement statistics

Crystallographic studies of rhGBA1

A co-crystal structure of rhGBA1 in complex with **ABP-IV** (MDW933) was obtained at 1.86 Å resolution to reveal a single molecule of **ABP-IV** bound covalently to the catalytic nucleophile (Glu340) of both rhGBA1 chains in the crystallographic dimer. Specifically, the cyclophellitol moiety reacts with Glu340 through its epoxide warhead, to form a covalent trans-diaxial ring opened cyclitol in the $^4C_1$ chair conformation (Fig. S2). Whilst only the reacted cyclitol and triazole linker of the ABP molecule bound in chain B could be modelled (likely due to disorder of the alkyl linker and/or probe decomposition), sufficient electron density was observed to model the full, intact probe bound in chain A. The C6-triazole linker and subsequent 4-carbon alkyl chain were modelled through a broad active site cleft, formed by Tyr244, Pro245, Phe246, Tyr313 and Asn396, which extends towards the dimer interface where the BODIPY tag binds (Fig. S3). The BODIPY binds in a hydrophobic cavity at the dimer interface formed by residues Leu241, Leu314, Phe316, Phe347 and Trp348 of rhGBA1 chain A and residues Leu241, Leu314, Phe316 and Leu317 of rhGBA1 chain B, consistent with the binding of a Cy5-tagged ABP we reported previously[15] and with a recently reported serendipitous co-complex of **ABP-IV** and *N*-acyl cyclophellitol aziridine (KY358).[16] Electron density was also observed for the BODIPY tag and the triazole-alkyl linker of a decomposed probe bound in a distant site on the surface of the TIM-barrel domain of rhGBA1 (S4).

Production and crystallization of rhGBA1

Recombinant human GBA1 (rhGBA1) was produced in an insect-baculovirus expression system and purified according to previously published procedures.[17] rhGBA1 was subsequently crystallised in a 48-well MRC sitting-drop vapour-diffusion format using previously reported conditions containing 0.2 µL GBA1 (10 mg.mL^{-1}) + 0.4 µL well solution [0.2 M sodium sulfate, 14% (v/v) PEG 3350, 0.25 M HEPES pH 7] + 0.1 µL seed solution (1:1000 dilution).[17]

ABP IV co-crystal complex

ABP IV was prepared at 50 mM in 100% DMSO and diluted to 5 mM in mother liquor containing 0.2 M sodium sulfate, 0.25 M HEPES pH 7.0, 14% (v/v) PEG 3350. Unliganded rhGBA1 crystals were soaked overnight in **ABP IV** spiked mother liquor before briefly transferring to a 25% (v/v) ethylene glycol cryoprotectant solution. Crystals were flash frozen in liquid N_2 for data collection.

Data collection, structure solution and refinement

Data were collected at the i03 beamline of the Diamond Light Source (DLS) UK and processed using XIA2[18] and AIMLESS[19,20] data reduction pipelines through the CCP4i2 suite.[21] The structure was solved by molecular replacement using MOLREP with the previously deposited unliganded structure (PDB 6TJK)[17] as homologous search model.

Structure refinement was performed using REFMAC[22] followed by several rounds of manual model building with COOT.[23,24] Idealized coordinate sets and refinement dictionaries for the ligand were generated using ACEDRG[25,26] in the CCP4 suite. Conformation of all sugars were validated using Privateer[27] and the structure was validated using MolProbity[28] and the wwPDB Validation service (validate-rcsb-1.wwpdb.org/) prior to deposition. All crystal structure figures were generated using CCP4 mg.[29] Data collection and refinement statistics are summarised in Table S3.

Crystallographic studies of rhGAA in complex with ABP-II

The structure of rhGAA (Myozyme®) soaked with **ABP-II** has been obtained at 1.9 Å resolution. As can be seen in Fig. S5, during the time-laps of rhGAA crystals bathing in crystallization solution supplemented with **ABP-II**, the rhGAA nucleophile Asp518 operated a nucleophilic attack on the *epi*-cyclophellitol aziridine warhead, leading to a covalent and irreversible enzyme-inhibitor complex where the cyclitol moiety of **ABP-II** adopts a $^{1}S_{3}$ chair conformation. The cyclitol hydroxyl groups establish the same hydrogen-bonding interactions as described for the rhGAA-*N*-PNT-DNM **15** complex in the main text. The nitrogen from the reacted aziridine establishes two water mediated contacts with Asp616 and Asp282, respectively. The alkyl chain is lined with Met519, Trp376, Trp481 and Phe525, and the triazole ring stacks against Phe525. Finally the subsequent alkyl chain blots against Phe525 and Val480 and the terminal nitrogen atom, last atom to be seen in the electron density map, establishes a hydrogen-bond interaction with the main-chain carbonyl of Val480. The remaining part of the probe, notably the TAMRA group, could not be observed in the electron density, most likely due to structural disorder. Globally, it appears that Phe525 is a major player in **ABP-II** recognition, where the ensemble of alkyl chains and the triazole adduct wrap around the side chain of this amino-acid. In summary it can be noticed that the **ABP-II** probe accommodates perfectly within the rhGAA substrate-binding groove, making it a suitable activity-based probe for GAA.

rhGAA sample preparation, crystallization and crystallographic procedures

Samples of recombinant human GAA (rhGAA) were kindly provided by the teams of Giancarlo Parenti and Marco Moracci, University of Naples. Shortly, these were residual amounts of the infusions of Myozyme® (Sanofi Genzyme, Cambridge, MA) administrated for the treatment of Pompe patients at the Department of Translational Medical Sciences, Federico II University, Naples, Italy. Sample conditioning and crystallization were performed as described

previously.[30] Crystal soaking was achieved by transferring crystals of rhGAA to small drops composed of crystallization liquor and either compound **15** or **ABP-II** at a final concentration of 10 mM, followed by incubation for ~3 hours. Crystals were then cryo-protected with reservoir solution supplemented with 30% (v/v) glycerol prior flash cooling in liquid N_2. X-ray diffraction data have been acquired at beam line Proxima2, Synchrotron Soleil, Gif-sur-Yvette, France, and processed with XDS[31] and the CCP4 software suite.[21] Structures were obtained by difference Fourier synthesis with REFMAC[22] using the native structure of rhGAA (PDB entry 5NN3) as starting model. Ligand coordinates were generated with jLigand.[32] Models were refined with subsequent rounds of Refmac[22] and Coot[24] respectively. Indices for the R_{free} cross-validation data sets were taken over from PDB entry 5NN4 and extended to 1.75 Å resolution for the complex with compound **15**. Model quality was assessed with internal modules of Coot[24] and with the Molprobity server.[28] Figures were generated with Pymol (The PyMOL Molecular Graphics System, Version 2.3.5, Schrödinger, LLC.).

3. NMR Spectra

^{1}H-NMR and ^{13}C-NMR spectra of 5'-TAMRA **ABP I** in MeOD

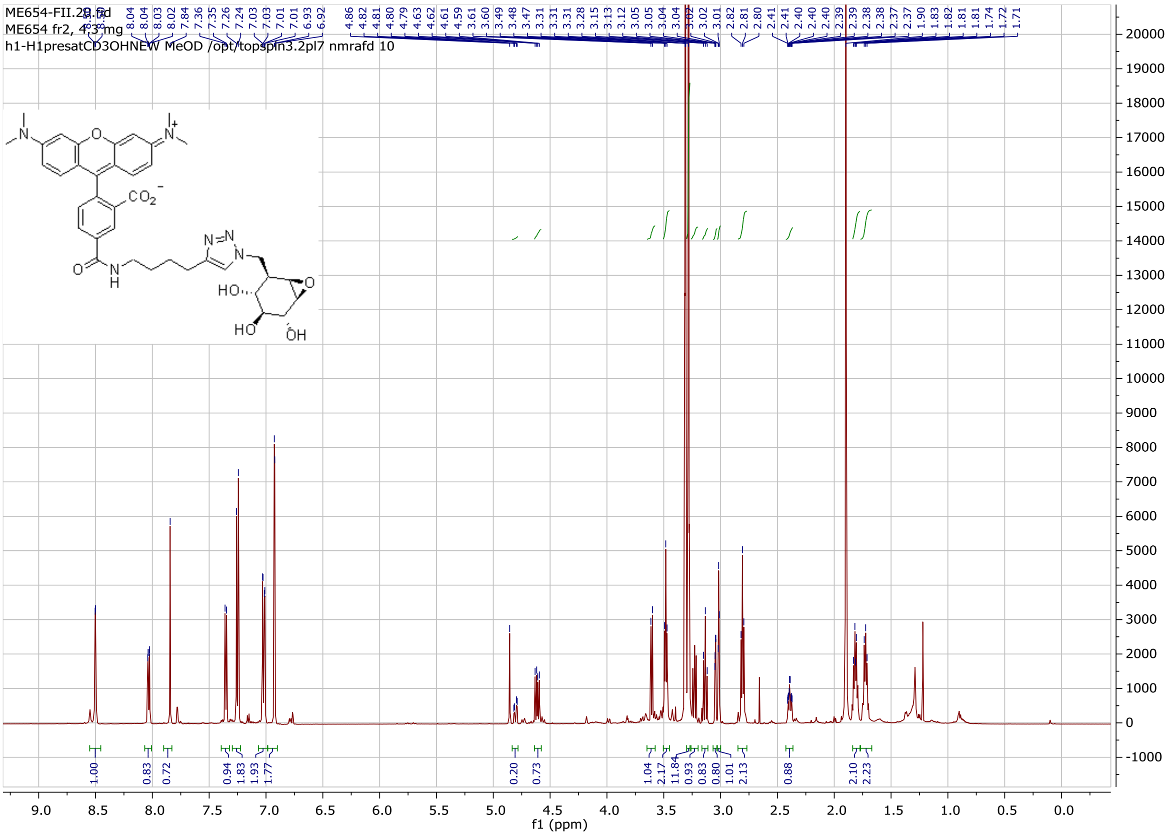

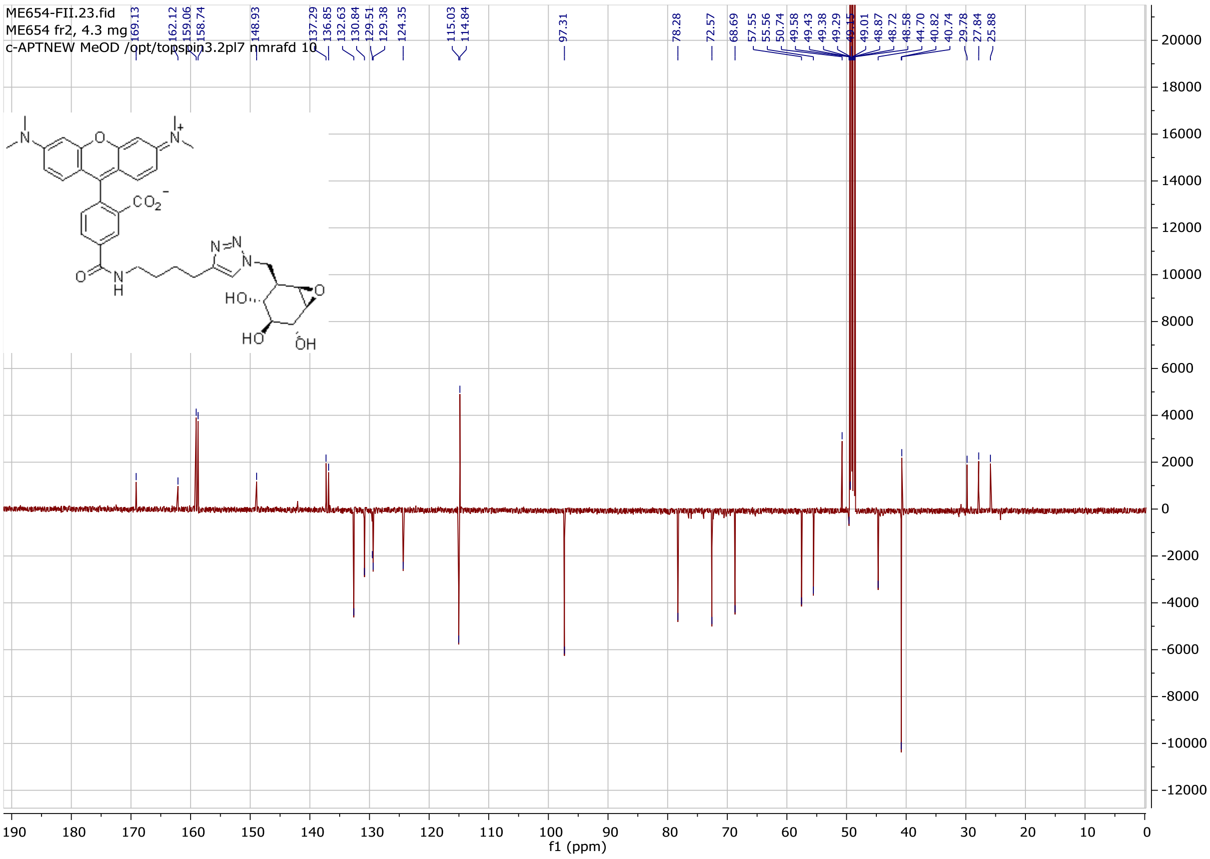

^1H-NMR and ^{13}C-NMR spectra of 6'-TAMRA **ABP I** in MeOD

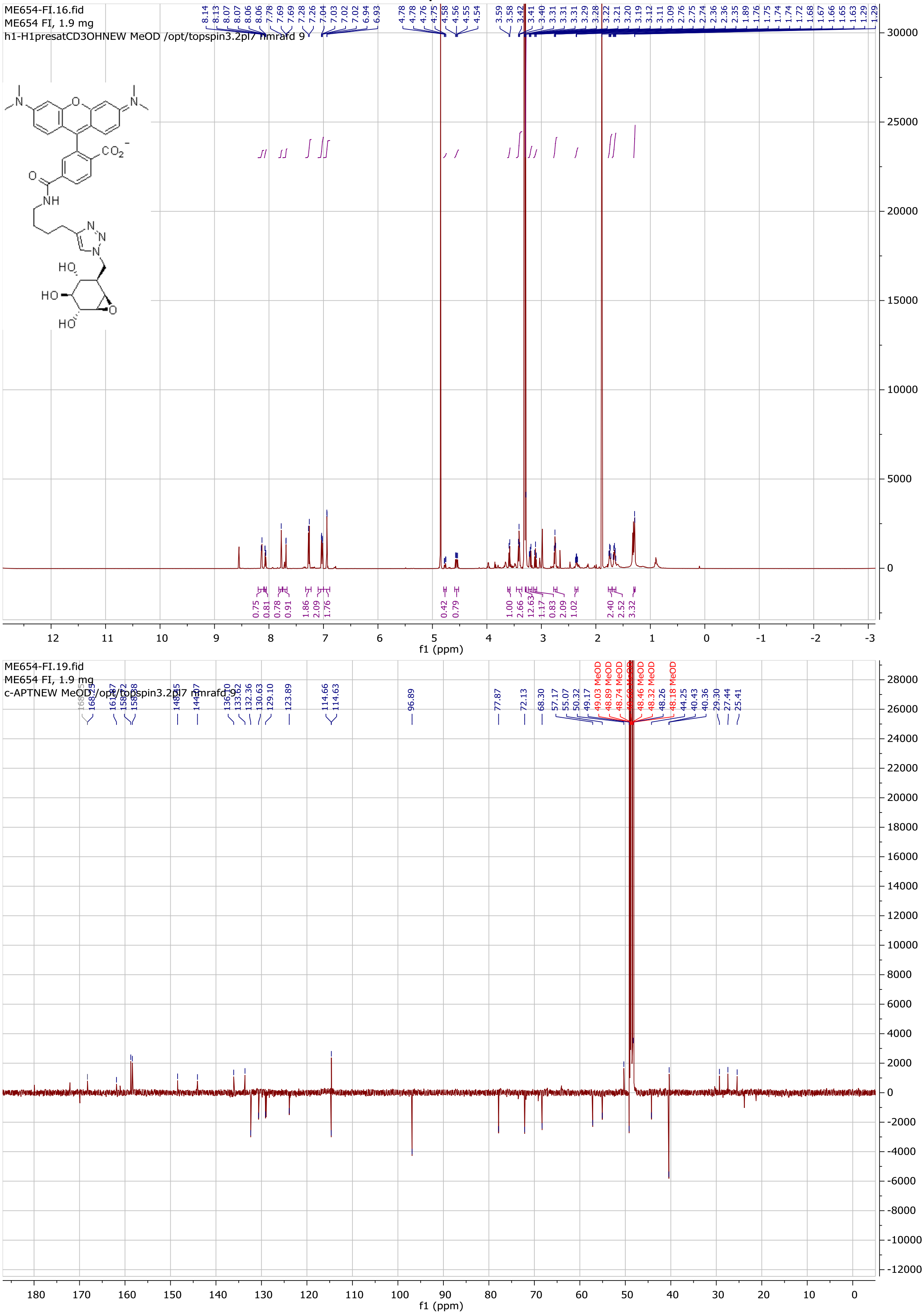

^{1}H-NMR and ^{13}C-NMR spectra of 5'- and 6'-TAMRA **ABP II** in MeOD

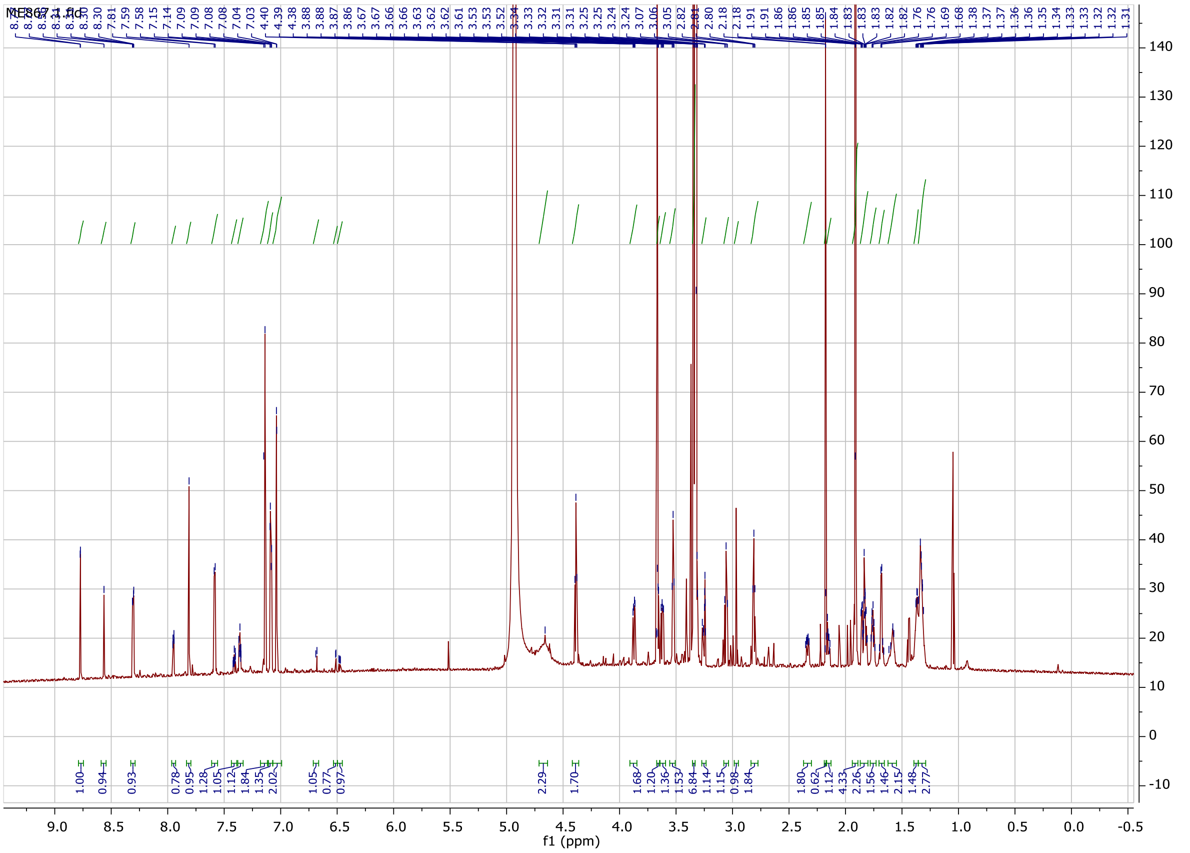

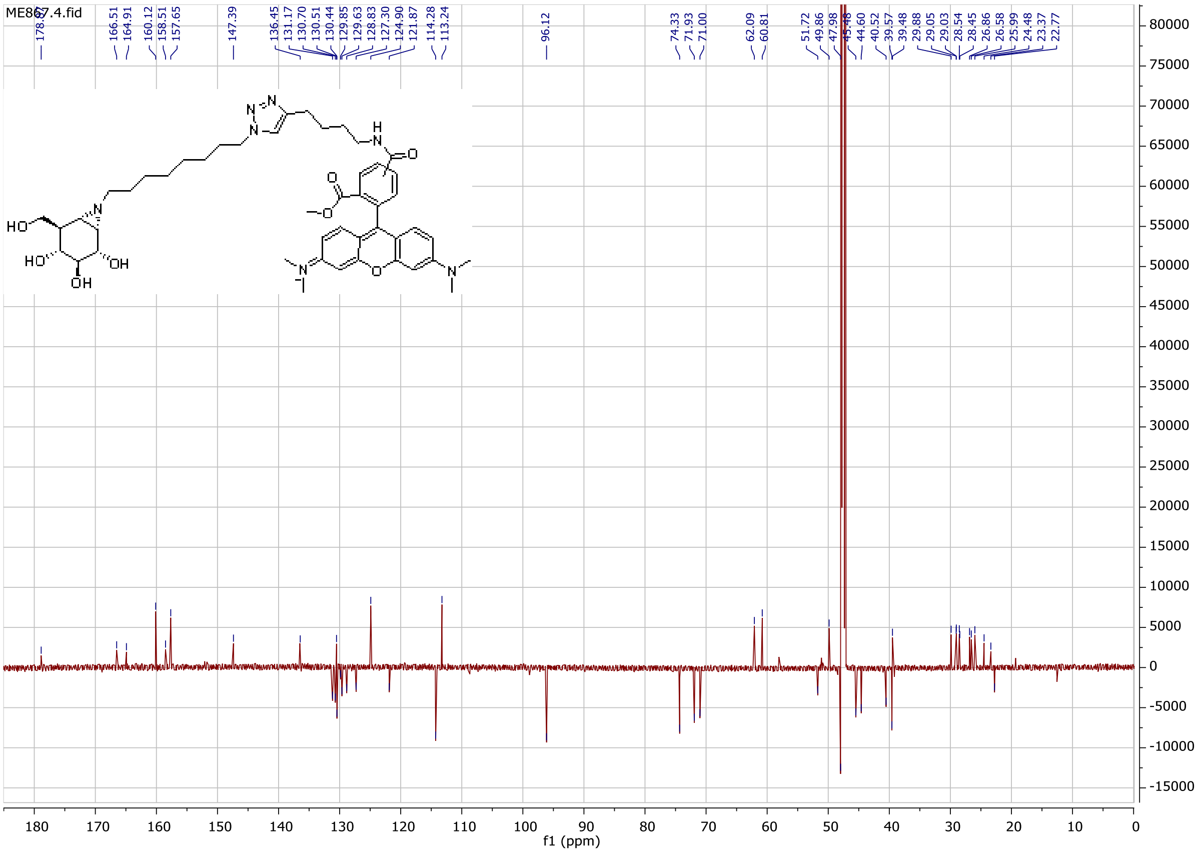

References

1 T. Wennekes, K. M. Bonger, K. Vogel, R. J. B. H. N. van den Berg, A. Strijland, W. E. Donker-Koopman, J. M. F. G. Aerts, G. A. van der Marel and H. S. Overkleeft, *European J. Org. Chem.*, 2012, 6420–6454.

2 K. M. Bonger, T. Wennekes, S. V. P. De Lavoir, D. Esposito, R. J. B. H. N. van den Berg, R. E. J. N. Litjens, G. A. van der Marel and H. S. Overkleeft, *QSAR Comb. Sci.*, 2006, **25**, 491–503.

3 A. Ghisaidoobe, P. Bikker, A. C. J. de Bruijn, F. D. Godschalk, E. Rogaar, M. C. Guijt, P. Hagens, J. M. Halma, S. M. Van't Hart, S. B. Luitjens, V. H. S. van Rixel, M. Wijzenbroek, T. Zweegers, W. E. Donker-Koopman, A. Strijland, R. Boot, G. van der Marel, H. S. Overkleeft, J. M. F. G. Aerts and R. J. B. H. N. van den Berg, *ACS Med. Chem. Lett.*, 2011, **2**, 119–123.

4 R. J. B. H. N. van den Berg, T. Wennekes, A. Ghisaidoobe, W. E. Donker-Koopman, A. Strijland, R. G. Boot, G. A. van der Marel, J. M. F. G. Aerts and H. S. Overkleeft, *ACS Med. Chem. Lett.*, 2011, **2**, 519–522.

5 D. Lahav, B. Liu, R. J. B. H. N. van den Berg, A. M. C. H. van den Nieuwendijk, T. Wennekes, A. T. Ghisaidoobe, I. Breen, M. J. Ferraz, C. L. Kuo, L. Wu, P. P. Geurink, H. Ovaa, G. A. van der Marel, M. van der Stelt, R. G. Boot, G. J. Davies, J. M. F. G. Aerts and H. S. Overkleeft, *J. Am. Chem. Soc.*, 2017, **139**, 14192–14197.

6 A. T. Ghisaidoobe, R. J. B. H. N. van Den Berg, S. S. Butt, A. Strijland, W. Donker-Koopman, S. Scheij, A. M. C. H. van den Nieuwendijk, G.-J. Koomen, A. van Loevezijn, M. Leemhuis, T. Wennekes, M. van der Stelt, G. van der Marel, C. A. A. van Boeckel, J. M. F. G. Aerts and H. S. Overkleeft, *J. Med. Chem.*, 2014, **57**, 9096–9104.

7 T. Wennekes, R. J. B. H. N. van den Berg, T. J. Boltje, W. E. Donker-Koopman, B. Kuijper, G. A. van der Marel, A. Strijland, C. P. Verhagen, J. M. F. G. Aerts and H. S. Overkleeft, *European J. Org. Chem.*, 2010, 1258–1283.

8 T. Wennekes, A. J. Meijer, A. K. Groen, R. G. Boot, J. E. Groener, M. van Eijk, R. Ottenhoff, N. Bijl, K. Ghauharali, H. Song, T. J. O'Shea, H. Liu, N. Yew, D. Copeland, R. J. van den Berg, G. A. van der Marel, H. S. Overkleeft and J. M. Aerts, *J. Med. Chem.*, 2010, **53**, 689–698.

9 T. Wennekes, R. J. B. H. N. van den Berg, K. M. Bonger, W. E. Donker-Koopman, A. Ghisaidoobe, G. A. van der Marel, A. Strijland, J. M. F. G. Aerts and H. S. Overkleeft, *Tetrahedron Asymmetry*, 2009, **20**, 836–846.

10 H. S. Overkleeft, G. H. Renkema, J. Neele, P. Vianello, I. O. Hung, A. Strijland, A. M. van der Burg, G. J. Koomen, U. K. Pandit and J. M. F. G. Aerts, *J. Biol. Chem.*, 1998, **273**, 26522–26527.

11 H. Takahata, Y. Banba, H. Ouchi and H. Nemoto, *Org. Lett.*, 2003, **5**, 2527–2529.

12 W. Chen, K. Zhao, G. Zhou and H. Nie, *Org. Biomol. Chem.*, 2016, **14**, 9466–9471.

13 A. L. Concia, C. Lozano, J. A. Castillo, T. Parella, J. Joglar and P. Clapés, *Chem. - A Eur. J.*, 2009, **15**, 3808–3816.

14 J. Jiang, W. W. Kallemeijn, D. W. Wright, A. M. C. H. van den Nieuwendijk, V. C. Rohde, E. C. Folch, H. van den Elst, B. I. Florea, S. Scheij, W. E. Donker-Koopman, M. Verhoek, N. Li, M. Schürmann, D. Mink, R. G. Boot, J. D. C. Codée, G. A. van der Marel, G. J. Davies, J. M. F. G. Aerts and H. S. Overkleeft, *Chem. Sci.*, 2015, **6**, 2782–2789.

15 M. Artola, C.-L. Kuo, L. Lelieveld, R. Rowland, G. van der Marel, J. Codée, R. Boot, G. Davies, J. Aerts and H. Overkleeft, *J. Am. Chem. Soc.*, 2019, **141**, 4214–4218.

16 G. J. Davies, R. J. Rowland, Y. Chen, I. Breen, L. Wu, W. A. Offen, T. Beenakker, Q. Su, A. M. C. H. van den Nieuwendijk, J. M. F. G. Aerts, M. Artola and H. S. Overkleeft, *Chem. – A Eur. J.*,

2021, **27**, 16377–16388.

17 R. J. Rowland, L. Wu, F. Liu and G. J. Davies, *Acta Crystallogr. Sect. D Struct. Biol.*, 2020, **76**, 565–580.

18 G. Winter, *J. Appl. Crystallogr.*, 2010, **43**, 186–190.

19 P. Evans, *Acta Crystallogr. Sect. D Biol. Crystallogr.*, 2006, **62**, 72–82.

20 P. R. Evans and G. N. Murshudov, *Acta Crystallogr. Sect. D Biol. Crystallogr.*, 2013, **69**, 1204–1214.

21 M. D. Winn, C. C. Ballard, K. D. Cowtan, E. J. Dodson, P. Emsley, P. R. Evans, R. M. Keegan, E. B. Krissinel, A. G. W. Leslie, A. McCoy, S. J. McNicholas, G. N. Murshudov, N. S. Pannu, E. A. Potterton, H. R. Powell, R. J. Read, A. Vagin and K. S. Wilson, *Acta Crystallogr. Sect. D Biol. Crystallogr.*, 2011, 67, 235–242.

22 O. Kovalevskiy, R. A. Nicholls, F. Long, A. Carlon and G. N. Murshudov, *Acta Crystallogr. Sect. D, Struct. Biol.*, 2018, **74**, 215–227.

23 P. Emsley, B. Lohkamp, W. G. Scott and K. Cowtan, *Acta Crystallogr. D. Biol. Crystallogr.*, 2010, **66**, 486–501.

24 A. Casañal, B. Lohkamp and P. Emsley, *Protein Sci.*, 2020, **29**, 1069–1078.

25 F. Long, R. A. Nicholls, P. Emsley, S. Gražulis, A. Merkys, A. Vaitkus and G. N. Murshudov, *Acta Crystallogr. Sect. D Struct. Biol.*, 2017, **73**, 112–122.

26 F. Long, R. A. Nicholls, P. Emsley, S. Gražulis, A. Merkys, A. Vaitkus and G. N. Murshudov, *Acta Crystallogr. Sect. D Struct. Biol.*, 2017, **73**, 103–111.

27 J. Agirre, J. Iglesias-Fernández, C. Rovira, G. J. Davies, K. S. Wilson and K. D. Cowtan, *Nat. Struct. Mol. Biol.*, 2015, 22, 833–834.

28 C. J. Williams, J. J. Headd, N. W. Moriarty, M. G. Prisant, L. L. Videau, L. N. Deis, V. Verma, D. A. Keedy, B. J. Hintze, V. B. Chen, S. Jain, S. M. Lewis, W. B. Arendall III, J. Snoeyink, P. D. Adams, S. C. Lovell, J. S. Richardson and D. C. Richardson, *Protein Sci.*, 2018, **27**, 293–315.

29 S. McNicholas, E. Potterton, K. S. Wilson and M. E. M. Noble, *Acta Crystallogr. Sect. D Biol. Crystallogr.*, 2011, **67**, 386–394.

30 V. Roig-Zamboni, B. Cobucci-Ponzano, R. Iacono, M. C. Ferrara, S. Germany, Y. Bourne, G. Parenti, M. Moracci and G. Sulzenbacher, *Nat. Commun.*, 2017, **8**, 1111.

31 W. Kabsch, *Acta Crystallogr. D. Biol. Crystallogr.*, 2010, **66**, 125–32.

32 A. A. Lebedev, P. Young, M. N. Isupov, O. V. Moroz, A. A. Vagin and G. N. Murshudov, *Acta Crystallogr. Sect. D Biol. Crystallogr.*, 2012, **68**, 431–440.
